# Integrating scRNA-seq and bulk RNA-seq to explore the differentiation mechanism of human nail stem cells mediated by onychofibroblasts

**DOI:** 10.3389/fcell.2024.1416780

**Published:** 2024-06-03

**Authors:** Xia Fang, Jiateng Zhou, Yating Yang, Dawei Li, Bin Wang

**Affiliations:** ^1^ Department of Plastic and Reconstructive Surgery, Shanghai Ninth People’s Hospital, Shanghai Jiao Tong University School of Medicine, Shanghai, China; ^2^ Department of Plastic Surgery, The Second Affiliated Hospital Zhejiang University School of Medicine, Hangzhou, China; ^3^ Department of Plastic Surgery, Huashan Hospital, Fudan University, Shanghai, China

**Keywords:** onychofibroblast, nail stem cell, differentiation, BMP4, TGF-beta pathway, transcriptomic profiles, digit regeneration

## Abstract

**Introduction:** Nail stem cell (NSC) differentiation plays a vital role in maintaining nail homeostasis and facilitating digit regeneration. Recently, onychofibroblasts (OFs), specialized mesenchymal cells beneath the nail matrix, have emerged as potential regulators of NSC differentiation. However, limited understanding of OFs’ cellular properties and transcriptomic profiles hinders our comprehension of their role. This study aims to characterize human OFs and investigate their involvement in NSC differentiation.

**Methods:** Human OFs were isolated and characterized for their mesenchymal stem cell (MSC)-like phenotype through flow cytometry and multilineage differentiation assays. Bulk RNA-seq analysis was conducted on three samples of OFs and control fibroblasts from human nail units to delineate their molecular features. Integrated analysis with scRNA-seq data was performed to identify key signaling pathways involved in OF-induced NSC differentiation. Co-culture experiments, siRNA transfection, RT-qPCR, and immunocytochemistry were employed to investigate the effect of OF-derived soluble proteins on NSC differentiation. Drug treatments, RT-qPCR, western blotting, and immunocytochemistry were used to verify the regulation of candidate signaling pathways on NSC differentiation *in vitro*.

**Results:** Human OFs exhibited slow cell cycle kinetics, expressed typical MSC markers, and demonstrated multilineage differentiation potential. Bulk RNA-seq analysis revealed differential gene expression in OFs compared to control fibroblasts, highlighting their role in coordinating nail development. Integrated analysis identified BMP4 as a pivotal signal for OFs to participate in NSC differentiation through mesenchymal-epithelial interactions, with the TGF-beta pathway possibly mediating this signal. OFs synthesized and secreted more BMP4 than control fibroblasts, and BMP4 derived from OFs induced NSC differentiation in a co-culture model. Recombinant human BMP4 activated the TGF-beta pathway in NSCs, leading to cell differentiation, while the BMP type I receptor inhibitor LDN193189 attenuated this effect.

**Discussion:** This study characterizes the cellular and molecular features of human OFs, demonstrating their ability to regulate NSC differentiation via the TGF-beta signaling pathway. These findings establish a connection between the dermal microenvironment and NSC differentiation, suggesting the potential of OFs, in conjunction with NSCs, for developing novel therapies targeting nail and digit defects, even severe limb amputation.

## Introduction

Nails are skin appendages that grow on the dorsal surface of digit tips. Despite their unassuming appearance, these mini-organs serve various functions, including providing physical protection for distal extremities and aiding in finger dexterity and fine manipulation (Saito et al., 2015). Recent research has discovered that the degree of nail preservation determines whether the digit tip can regenerate after amputation, both in humans and mice (Illingworth, 1974; Neufeld and Zhao, 1995). This remarkable attribute of nails is intimately linked to nail stem cells (NSCs) and their signaling pathways governing differentiation (Takeo et al., 2013; Lehoczky and Tabin, 2015). NSCs, residing in the nail epithelium, are dynamic adult stem cells capable of self-renewal and differentiation into requisite cell types (Naveau et al., 2014). Understanding the signaling cascades dictating NSC differentiation is crucial for unraveling the intricacies of digit regeneration.

The development of ectodermal appendages, including initiation, morphogenesis, and differentiation, is orchestrated by sequential mesenchymal-epithelial interactions (Naveau et al., 2014). For instance, dermal papilla cells mediate the proliferation and differentiation of hair follicle stem cells (Lee et al., 2018). Lee et al. coined the term “onychodermis” to designate the dermis beneath the nail matrix and proximal nail bed, characterized by the expression of CD10, CD13, and VCAN, thereby distinguishing it from adjacent mesenchymal tissues (Lee et al., 2006; Lee et al., 2012; Park et al., 2017). They postulated that fibroblasts within the onychodermis, termed onychofibroblasts (OFs), may mirror the functions of dermal papilla cells and are indispensable for nail development (Lee et al., 2022). However, research into the attributes of human OFs and their regulatory effect on NSC differentiation remains scarce due to limited accessibility to human nail specimens.

This study delves into the cellular properties of human OFs and analyzes their transcriptome profiles through the integration of scRNA-seq and bulk RNA-seq. Our findings highlight the role of BMP4 secreted by OFs as a pivotal mediator of mesenchymal-epithelial interaction, activating the TGF-beta pathway in NSCs to induce stem cell differentiation. Our research contributes to the comprehensive characterization of human OFs and NSCs, substantiating the necessity of BMP signaling for human NSC differentiation. Furthermore, it provides insights into nail and digit regeneration, offering potential avenues for advancements in this field.

## Materials and methods

### Human nail sample collection

Human nail samples were obtained from patients who underwent duplicated thumb excision. Written informed consent was obtained from patients’ guardians before sample collection following the Declaration of Helsinki and with approval from the Human Research Ethics Committee of Shanghai Ninth People’s Hospital (IRB number: SH9H-2021-T391-2). Detailed patient information is provided in Supplementary Table S1.

### Isolation and cell culture of nail stem cells

Nail samples were dissected within 2 h after the digit removal. Tissues were prepared into two parts: nail matrix and proximal nail fold. For the nail stem cells, tissues were minced and enzymatically dissociated with 2U/mL dispase Ⅱ (Roche) and 0.2 mg/mL DNase Ⅰ (Sigma) incubation for 2 h at 37°C. The collected suspension was centrifuged at 500 *g* and 4°C for 5 min. After discarding the supernatant, cells were stored on ice. Tissues were then transferred into 0.25% Trypsin (Gibco) solution and incubated at 37°C for 15 min. The dissociated cell suspension was collected and centrifuged at 500 *g* and 4°C for 5 min. Cell pellets obtained in the first and second rounds were washed with PBS (Gibco) and strained with a 70-μM filter (Falcon), centrifuged, and resuspended in DermaCult™ Keratinocyte Expansion Medium (STEMCELL Technologies). The primary nail epidermal cells were plated onto type IV collagen (Corning) coated dishes. Nail stem cells were selected according to their ability to adhere within 10 min at 37°C, and non-adherent cells were rinsed off gently as previous reports described (Papini et al., 2003; Kim et al., 2004). Cells were cultured at 37 °C, 5% CO2 incubator, and passaged at 80%–90% confluence. The DermaCult™ Keratinocyte Expansion Medium was changed every 3 days. Cells at passage three were used in the experiments as needed.

### Isolation and cell culture of onychofibroblasts and dermal fibroblasts

After harvesting the nail epidermal cells, the residual tissue of the nail matrix and proximal nail fold was separately washed in PBS and digested in Mesenchymal Stem Cell Medium (ScienCell) containing 0.2% collagenase NB 4 (Nordmark) at 37 C for 2 h. The cell suspension was strained with a 70-μm filter. The filtrate was centrifuged at 500 *g* for 5 min at RT, and the supernatant was discarded. The cell pellet was washed and centrifuged in PBS. After being resuspended in the Mesenchymal Stem Cell Medium, cells were plated onto dishes and cultured at 37 C, 5% CO2 incubator, and passaged at 80%–90% confluence. The medium was changed every 3 days. Cells at passage three were used in the experiments as needed.

### Histological staining

The surgically removed human nail tissue samples were fixed in 4% paraformaldehyde. After dehydration and paraffin embedding, the tissue sections were stained with hematoxylin and eosin, and Masson’s trichrome. The sections were sealed with a neutral resin and scanned with a Nikon Eclipse 90i digital camera under an optical microscope (Nikon).

### Cell surface markers staining and flow cytometry

Cells were harvested, washed, and re-suspended in the buffer (PBS containing 2% FBS). Approximately 1 × 10^5^ cells in 200 μL of buffer were incubated with fluorescein–conjugated antibodies for 30 min on ice in the dark, and pipet the cells gently every 10 min. Then add 1 mL of buffer to each sample, resuspend, and centrifuge the cells at 500 *g* × 5 min at 4 °C. For those unconjugated primary antibodies, cells need to be incubated with fluorescein-conjugated secondary antibodies and washed again. Finally, the cells were re-suspended in 200 μL of buffer, and the expression of cell surface markers was analyzed by flow cytometry. The antibodies used are listed in Supplementary Table S2.

### Cell cycle analysis

A total of 2 × 10^5^ cells were collected and fixed overnight in 70% ethanol at 4°C. The fixed cells were centrifuged and washed three times with PBS, then resuspended in 500 μL of PI/RNase staining buffer (BD Pharmingen) and incubated in the dark at RT for 30 min. Cells were strained with a 40-μm filter and analyzed by flow cytometry (Beckman Coulter) and FlowJo software (BD Biosciences) using the Dean–Jett–Fox model.

### Adipogenic differentiation

Adipogenic differentiation was conducted following the manufacturer’s instruction (Cyagen). Briefly, cells were seeded in the growth medium at a density of 1  ×  10^5^ per well in a 12-well plate. When cells grow to 100% confluence, the medium was then changed to adipogenic differentiation medium A (induction medium) for 3 days and then the medium was replaced by adipogenic differentiation medium B (maintenance medium). After 24  h, the medium was changed back to adipogenic differentiation medium A. Repeat the induction and maintenance process until sufficient lipid droplets of appropriate size appear, and then prepare for staining. As for the RT-qPCR test of adipogenic marker genes, RNA was extracted after 7 days of induction.

### Osteogenic differentiation

Osteogenic differentiation was conducted following the manufacturer’s instruction (Cyagen). Briefly, cells were seeded in the growth medium at a density of 1  ×  10^5^ per well in a 12-well plate. When cells grow to 70% confluence, the growth medium was then changed to an osteogenic differentiation medium. The osteogenic medium was changed every 3 days. About 3 weeks after induction, Alizarin red staining was performed. As for the RT-qPCR test of osteogenic marker genes, RNA was extracted after 7 days of induction.

### Chondrogenic differentiation

Chondrogenic differentiation was conducted following the manufacturer’s instruction (Cyagen). Briefly, 3  × 10^5^ cells were transferred into a 15 mL centrifuge tube and centrifuged at 250 *g* for 4 min. After aspirating off the supernatant, 0.5 mL of chondrogenic differentiation premix medium was added to resuspend the cell pellet. Then cells were centrifuged at 150 *g* for 5 min. Repeat this step to wash the cells again. Resuspend cells in a complete chondrogenic differentiation medium (supplement: premix is 1:100) and continue centrifugation at 150 *g* for 5 min. Loosen the centrifuge tube cover for gas exchange. Place it vertically in a 37 C, 5% CO_2_ incubator. After 48 h, the cells gathered to form chondrocytes, which could be suspended in the liquid by gently tapping the bottom of the centrifuge tube. The chondrogenic medium was changed every 3 days until the diameter of the chondrosphere reached 1.5 mm and then prepared for staining. As for the RT-qPCR test of chondrogenic marker genes, RNA was extracted after 7 days of induction.

### Oil red O and alizarin red staining

The differentiation medium was removed. Cells were rinsed with PBS and fixed with 4% PFA for 30 min at RT. Then cells were rinsed twice with PBS and stained with 1 mL of working solution. For oil red O staining, cells were stained for 30 min at RT. For alizarin red staining, cells were stained for 10 min at RT. After removing the staining solution, rinse the wells again with PBS three times. Cells were visualized and analyzed under a microscope.

### Alcian blue staining

The differentiation medium was removed. Chondrospheres were rinsed with PBS and fixed with 4% PFA for 30 min at RT. After dehydration and paraffin embedding, they were sectioned at 3 μm. The sections were dewaxed and rehydrated with 95%, 85%, 70%, 50% alcohol. After drying by air, sections were stained with alcian blue solution at 37 C for 1 h. Then sections were rinsed with running water for 5 min, air dried, and observed under a microscope.

### RNA extraction and RT-qPCR

Total RNA from the cells was extracted using TRIzol reagent (Invitrogen), and the quality and concentration were measured by Nanodrop (Thermo Fischer Scientific). 1 μg total RNA was reverse transcribed using 4×Reverse Transcription Master Mix (Ezbioscience). The qPCR was then performed using 2× SYBR Green qPCR Master Mix (Ezbioscience), on an ABI QuantStudio 6 Flex System. The thermal cycling of the qPCR reaction was started with a predenaturation step at 95 C for 5 min and followed by 40 cycles of amplification (denaturation at 95 C for 10 s, annealing and extension at 60 C for 30 s). The expression level of mRNA was normalized using the level of 18S and the relative fold changes were calculated using the 2^−ΔΔCт^ method. The primers sequences are listed in Supplementary Table S3.

### Immunofluorescence and immunocytochemistry

For immunofluorescence, surgical nail samples were sectioned parallel to the longitudinal nail plate direction. They were formalin-fixed, embedded in paraffin, and sectioned serially at 5 μm. Sections underwent dewaxing and rehydration through xylene and ethanol treatment. Antigen retrieval was performed using heat and citrate buffer, pH 6.0 (Beyotime). Sections were washed in PBS and permeabilized with 0.1% Triton X-100 (Sigma) (dissolved in PBS) at RT for 20 min. Following three washes with PBS, sections were blocked in 10% donkey serum (Solarbio) at RT for 1 h. For immunocytochemistry, cells were seeded on coverslips and fixed in 4% paraformaldehyde at RT for 15 min, and then permeabilized and blocked. The sections/coverslips were incubated with primary antibodies overnight at 4 °C. Then they were washed with PBS containing 0.1% Tween-20 (Sigma) (PBST) and incubated with fluorescein–conjugated secondary antibodies for 1 h at RT. Sections/coverslips were then washed again with PBST, mounted with DAPI containing-mounting medium (Abcam), and examined using a fluorescence microscope (Lecia). Quantify the mean fluorescence intensity of captured images using ImageJ. The primary and secondary antibodies used are listed in Supplementary Table S2.

### Protein extraction and western blotting

Cells were lysed by RIPA buffer (Beyotime) containing 1 mM PMSF (Beyotime) for 10 min on ice followed by centrifugation at 12,000 *g* for 5 min at 4 C. The supernatant was collected, and the concentration was quantified using the BCA Protein Assay Kit (TaKaRa). The protein samples were mixed with 5× loading buffer (Epizyme), and then heated at 99 C for 5 min. The proteins (10 μg per sample) were separated by SDS-PAGE and transferred onto polyvinylidene fluoride membranes (Roche). After blocking with 5% BSA dissolved in TBST (tris-buffered saline-Tween 20) at RT for 1 h, the membranes were incubated with primary antibodies at 4 C overnight. After washing three times in TBST for 5 min/wash, the membranes were incubated with horseradish peroxidase-conjugated secondary antibody for 1 h at RT. The signals were detected by electrochemiluminescence reagent (Invitrogen). Protein bands were visualized in MiniChemi 610 Plus system (Sinsage). The blot images were quantified by densitometry using ImageJ. The primary and secondary antibodies used are listed in Supplementary Table S2.

### Enzyme-linked immunosorbent assay

Cells were seeded in 6-well plates at a density of 2  ×  10^5^ cells for 72 h. Then the cell supernatant from different samples was collected and analyzed using a human BMP4 ELISA kit (R&D Systems) following the manufacturer’s protocol. Briefly, 100 µL of assay diluent was added to each well. 50 μL of standard, medium control, and medium samples were added to each well. After covering with a plate sealer, the plate was incubated at RT for 2 h. Then aspirate each well and wash, repeating the process 3 times for a total of four washes. Add 200 µL of conjugate to each well, cover with a new plate sealer, and incubate in the dark at RT for 2 h. Aspirate and wash 4 times again. Add 200 µL substrate solution to each well. After incubating in the dark at RT for 30 min, 50 µL of stop solution was added to each well. The absorbance was measured at 450 nm (PerkinElmer).

### Cell clone staining

1,000 cells were seeded in a 10 cm dish at low density. The medium was freshed every 3 days, and on day 14, cells were fixed for 30 min with 4% PFA. After staining with 0.05% crystal violet (Servicebio), the clones became visible to the naked eye.

### Indirect co-culture

1 × 10^5^ NSCs were seeded in the lower chamber of a transwell culture inset (0.4 μm pore size, Corning). Then 1 × 10^5^ untreated or *BMP4 knockdown* onychofibroblasts were seeded in the upper chamber in the induced group, while the control group only added the culture medium. After 3 days of co-culture, the cells in the lower chamber were collected for RT-qPCR or ICC.

### RNA interference

The BMP4 siRNAs and scrambled negative control were purchased from RiboBio Co., Ltd (Cat.no PA20240129014). For siRNA transfection of onychofibroblasts, Lipofectamine RNAiMAX was used according to the manufacturer’s protocol (Thermo Fisher Scientific).

### Cell counting kit-8 assays

Cells were seeded in triplicate in 96-well plates at a density of ∼4,000 cells per well. Recombinant human BMP-4 (PeproTech) was applied at 10, 20, 50, and 100 ng/mL after cell adhesion. After incubation for 24, 48, 72, 96, and 120 h, a cell counting kit-8 (Dojindo) was used for cell viability assay, following the manufacturer’s instructions. The absorbance was measured at 450 nm (PerkinElmer).

### Bulk RNA sequencing and data analysis

For bulk RNA-seq analysis, total RNA was extracted from isolated human onychofibroblasts (n = 3) and dermal fibroblasts derived from proximal nail fold (n = 3) using a TRIzol kit (Invitrogen), according to the manufacturer’s instructions. RNA quality was evaluated using Agilent Bioanalyzer 2,100 (Agilent Technologies) and examined using RNase-free agarose gel electrophoresis. Complementary DNA library preparation and sequencing were performed according to Illumina standard protocol. Sequencing quality analysis of the raw data was performed using FASTQC software (http://www.bioinformatics.babraham.ac.uk/projects/fastqc). Per base sequence quality, per sequence quality scores, per base sequence content, and sequence length distribution all indicated that the data quality was high (Supplementary QC. zip). Gene expression analysis of identified transcripts was performed using the DESeq2 package in R software. The raw data of gene expression profiles have been submitted to the GEO database (https://www.ncbi.nlm.nih.gov/geo/), and the Geo accession number is GSE263876. Genes/transcripts with *p*-value [false discovery rate (FDR)] < 0.05, and |fold change| > 1.5 were considered to be differentially expressed genes (DEGs). We performed principal component analysis (PCA) to reveal the structure or relationship of the samples using R software. DEGs were annotated using Gene Ontology (GO) and the Kyoto Encyclopedia of Genes and Genomes (KEGG) databases. Specifically, biological processes and associated cellular pathways enrichment were assessed for the DEGs in the OF vs DF. In addition, the JASPAR database was used to predict the potential transcription factor binding sites (TFBS).

### scRNA-seq data reanalysis

The scRNA-seq data used in the current study is publicly available with the GEO database (GSE158970). The raw gene expression matrix was filtered, normalized using the Seurat package in R software, and selected according to the following criteria: 200 < features <10,000; and ≤30% of mitochondrial gene expression in UMI counts. A total of 11,621 remaining cells were enrolled in the final analysis. The three single-cell datasets were integrated and corrected for batch effects. The integrated data were used for graph-based clustering and visualization with the Seurat R package. PCA and Uniform Manifold Approximation and Projection (UMAP) analysis were used for the single cell-to-cell relation description. DEGs for each cluster were identified using FindAllMarkers () function in the Seurat R package with default parameters. Cell clusters were then annotated following the annotation of the original research and a literature review. For the second-level clustering, we isolated the fibroblast and keratinocyte clusters in the broad cell type UMAP, respectively. We further subclustered these cell types by reapplying the Seurat R package and identified the DEGs. To characterize the relative activation of signaling pathways in various nail keratinocyte populations, gene set enrichment analysis was performed using the R package QuSAGE as described previously to achieve the enrichment status and enrich the significance of each gene set.

### Statistical analysis

All values are presented as the mean ± standard deviation (SD). The results were analyzed using GraphPad Prism 9 (GraphPad Software Inc.). Unless otherwise indicated, Student’s t-test (two-tailed) or one-way ANOVA was used to analyze the difference between two or multiple groups, respectively. Differences were considered significant at *p* < 0.05.

## Results

### Human onychofibroblasts exhibit mesenchymal stem cell-like properties

**FIGURE 1:**
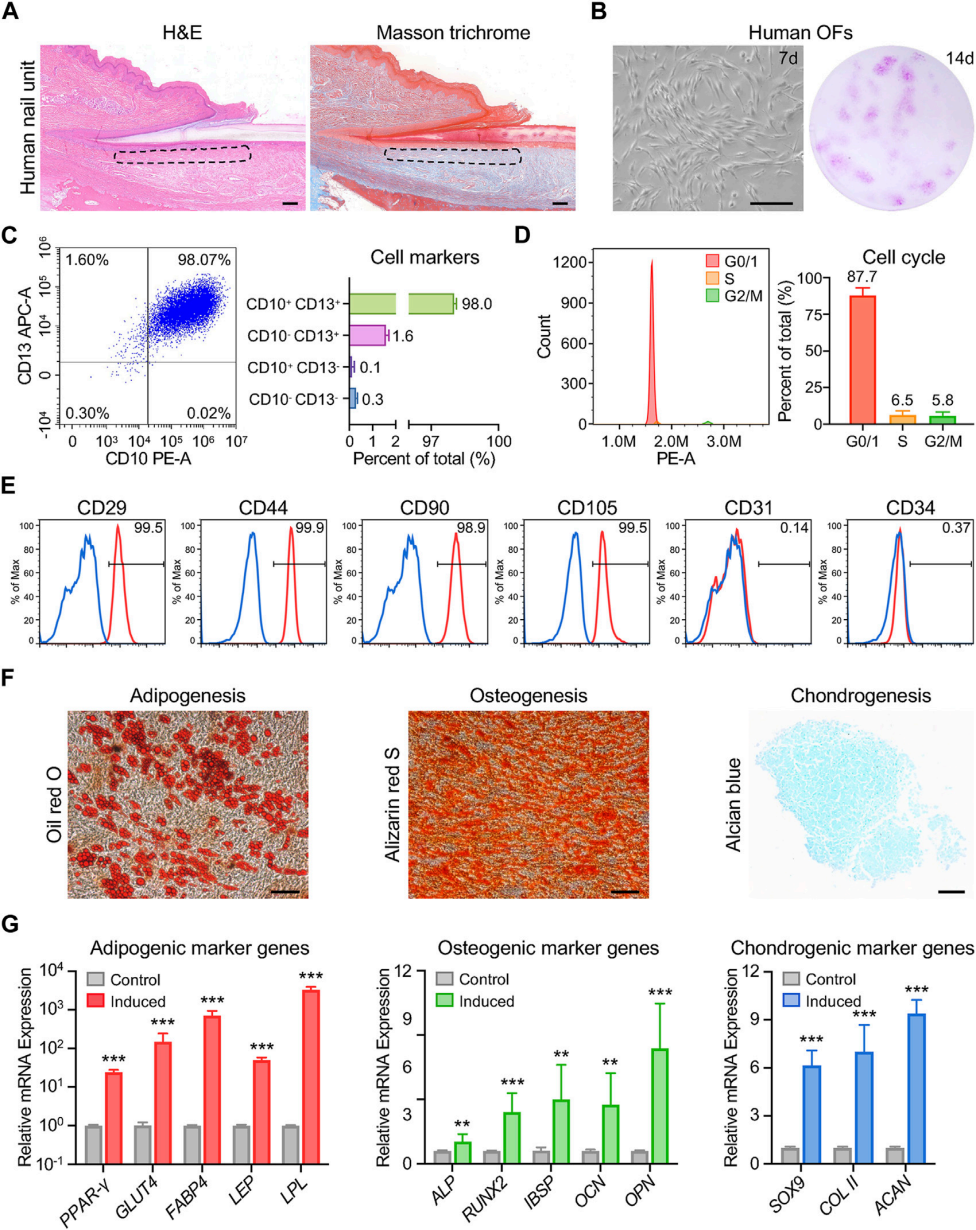
Characterization of human onychofibroblasts: cellular and molecular analysis. **(A)** H&E and Masson trichrome staining of the human nail unit. The dotted area indicates the location of the onychodermis. **(B)** *In vitro* culture showing the cell morphology and colony formation of human OFs. **(C)** Flow cytometric analysis showing the expression of CD10 and CD13 in human OFs. **(D)** Cell cycle analysis of human OFs. **(E)** Flow cytometric analysis demonstrating the expression of typical MSC markers in human OFs. **(F)** Cell morphology and staining results of human OFs after differentiation induction. **(G)** Relative mRNA expression levels of differentiation-related genes in human OFs after induction. Scale bars, 100 μm. The results presented are the mean ± SD of three biologically independent replicates. **p < 0.01, ***p < 0.001.

Nails and hair, both being skin appendages, exhibit highly keratinized structures. A recent study suggested a similarity in transcriptional expression profiles between the onychodermis and dermal papilla (Shim et al., 2022). Intrigued by this finding, we aimed to investigate whether human onychofibroblasts (OFs) shared properties akin to dermal papilla cells, such as a mesenchymal stem cell (MSC)-like phenotype (Dominici et al., 2006; Wang et al., 2020; Hoogduijn et al., 2006). We harvested human onychodermis samples from the nail root to the lunula (Figure 1A). Following enzymatic dissociation and *in vitro* expansion in conditioned medium, OFs were isolated, which were adherent fibroblast-like cells with the ability to form colonies (Figure 1B). Approximately 98.0% of OFs were positive for CD10 and CD13 as previously described (Figure 1C; Supplementary Figure S1). Furthermore, nearly 87.7% of OFs were found in the G0/1 phase (Figure 1D), indicative of their slow cell cycle kinetics resembling that of MSCs. Flow cytometric analysis demonstrated the expression of typical MSC markers, including CD29, CD44, CD90, and CD105, along with the absence of CD31 and CD34 in OFs (Figure 1E). We proceeded to evaluate the multipotentiality of OFs *in vitro*. Oil red O staining revealed numerous lipid droplet vacuoles following adipogenic induction, while Alizarin red staining indicated the formation of calcified nodules, and Alcian blue staining revealed the presence of Aizen proteoglycans (Figure 1F). Additionally, the relative expression levels of adipogenic genes (*PPAR-γ*, *GLUT4*, *FABP4*, *LEP*, *LPL*), osteogenic genes (*ALP*, *RUNX2*, *IBSP*, *OCN*, *OPN*), and chondrogenic genes (*SOX9*, *COL II*, *ACAN*) were upregulated upon *in vitro* induction (Figure 1G). These findings suggest that OFs exhibit MSC-like characteristics and possess the potential to differentiate into adipogenic, osteogenic, and chondrogenic lineages. In summary, human onychofibroblasts are specialized mesenchymal cells within the nail unit, displaying a stem cell-like phenotype, and potentially playing a significant role in maintaining nail homeostasis.

### Unique transcriptomic signatures of human onychofibroblasts reveal their role in nail development

**FIGURE 2:**
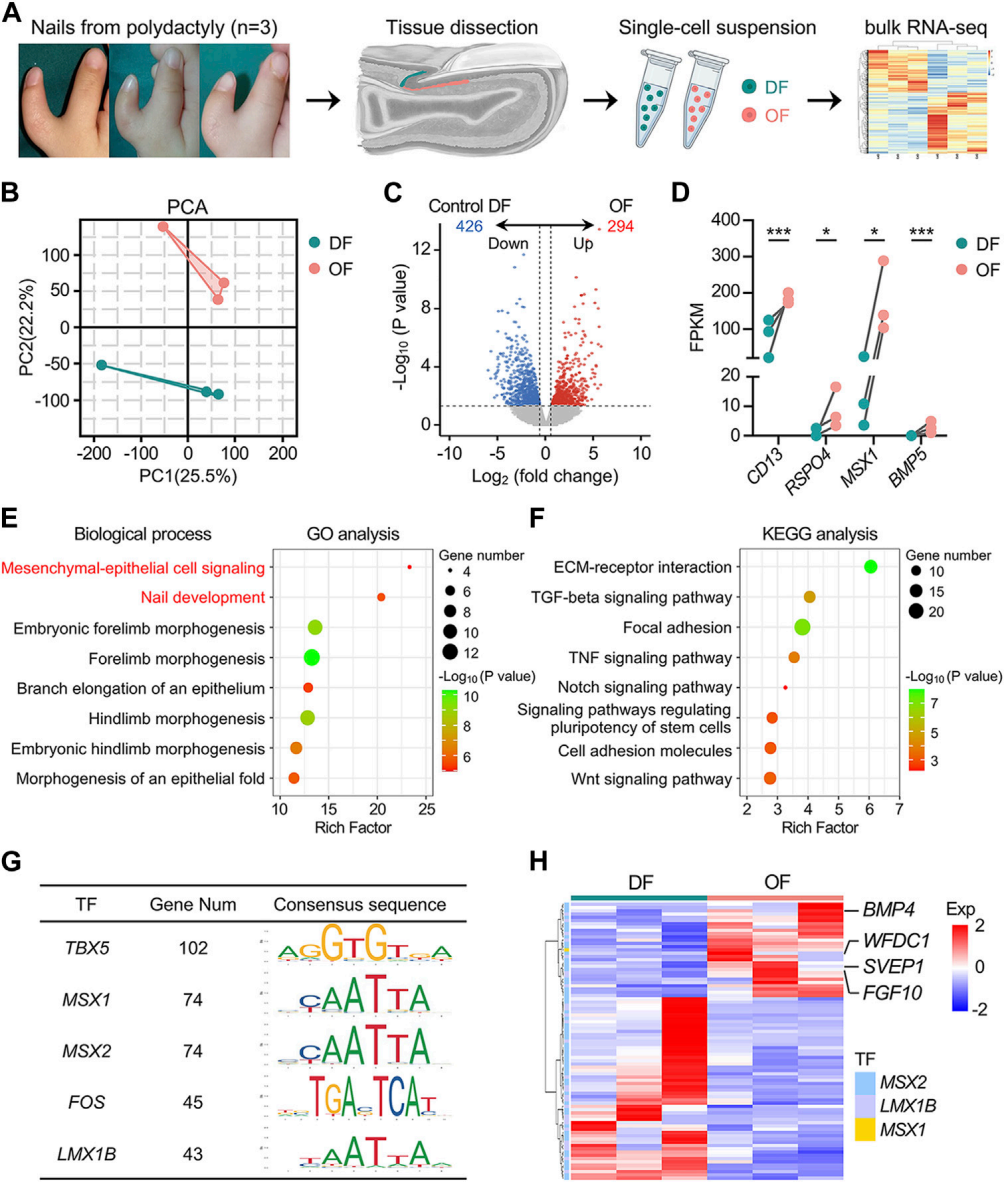
Transcriptomic analysis reveals the unique role of onychofibroblasts in human nail development. **(A)** Experimental flowchart. This schematic diagram outlines the process of specimen collection, primary cell isolation, and bulk RNA sequencing. **(B)** PCA analysis. Percentages on the PCA axis indicate the proportional variance explained by each principal component. **(C)** Volcano plot illustrating the DEGs between DF and OF groups, with criteria set as *p* < 0.05 and |fold change| > 1.5. Red indicates upregulation, and blue indicates downregulation. **(D)** Expression of four reported genes in the two groups. The *y*-axis represents FPKM values. **(E)** GO analysis and **(F)** KEGG analysis of DEGs between DF and OF groups. Bubble size represents gene number, color depth indicates the *p*-value, and the rich ratio represents the gene number/total gene number on the *y*-axis. **(G)** Top five promising TFs predicted by TFBSs analysis. **(H)** Heatmap illustrating the expression of target genes of *MSX2*, *LMX1B*, and *MSX1*, with genes encoding extracellular proteins listed on the right panel. Exp, expression.

To characterize human OFs in more detail, we conducted bulk RNA sequencing on OFs and dermal fibroblasts adjacent to the proximal nail fold (DFs) (Figure 2A). We obtained high-quality transcriptome of six samples (3 vs. 3) (Supplementary QC. zip). Principal component analysis (PCA) projection revealed significant separation in the PC space, indicating substantial differences in gene expression between OFs and DFs (Figure 2B). Compared to DFs, 294 genes were upregulated, and 426 genes were downregulated in OFs (Figure 2C). Notably, *CD13*, *RSPO4*, *MSX1,* and *BMP5* exhibited higher expression in OFs, consistent with previous reports (Figure 2D). Gene Ontology (GO) analysis revealed enrichment of genes involved in mesenchymal-epithelial cell signaling and nail development in OFs, affirming their role in nail growth (Figure 2E). Furthermore, KEGG pathway analysis highlighted upregulation of various components of the TGF-beta and Wnt signaling pathways in OFs, including *BMP4*, *BMP5*, *WNT2B*, and *RSPO4*, suggesting their potential to activate responsive cells via secretion of these proteins (Figure 2F and Supplementary Figure S2A,B). Additionally, several upregulated genes in OFs showed promise in regulating stem cell pluripotency (Figure 2F; Supplementary Figure S2C). Meanwhile, fibroblasts play a crucial role in extracellular matrix (ECM) production and remodeling. Our sequencing results revealed upregulation of numerous transcripts related to ECM-receptor interaction and focal adhesion pathways in OFs (Figure 2F and Supplementary Figures S2D,E). Further analysis predicted 17 transcription factors (TFs) as upstream regulators of differentially expressed genes (DEGs) (Supplementary Figure S3A). Among them, *TBX5*, *MSX1*, *MSX2*, *FOS*, and *LMX1B* were identified as the top five promising hubs, supported by transcription factor binding sites (TFBSs) analysis, and their elevated expression in OFs was validated through RT-qPCR (Figure 2G and Supplementary Figure S3A–C). Given the association of mutations in *MSX1*, *MSX2*, and *LMX1B* with nail development disorders, we focused on target genes encoding extracellular proteins of these TFs. Such proteins may serve as soluble mediators involved in signaling from OFs to NSCs. Thus, *BMP4*, *FGF10*, *WFDC1*, and *SVEP1* were found to be potential factors that may play a role (Figure 2H). These analyses underscore the distinct transcriptional profiles of OFs compared to DFs, indicative of their specialized role in coordinating nail development. Our preliminary identification of signaling molecules secreted by human OFs holds promise for further elucidating the differentiation mechanism of human NSCs.

### Human onychofibroblasts govern nail stem cell differentiation via BMP4

**FIGURE 3:**
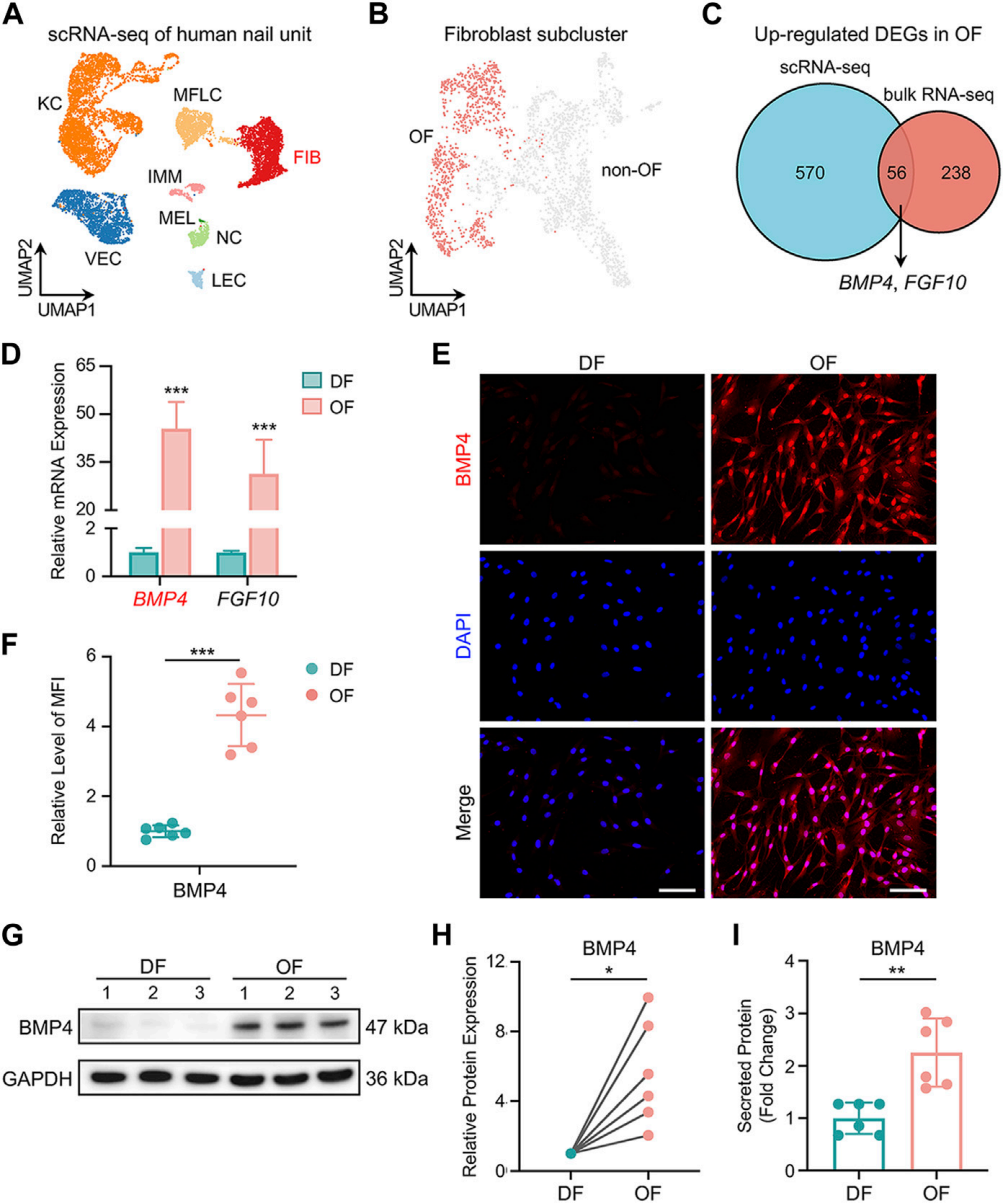
Expression of BMP4 in human onychofibroblasts. **(A)** Major cell types in the human nail unit revealed by scRNA-seq. KC, keratinocyte; FIB, fibroblast; MFLC, myofibroblast-like cell; VEC, vascular endothelial cell; LEC, lymphatic endothelial cell; IMM, immune cell; NC, neural cell; MEL, melanocyte. **(B)** UMAP visualization of the human OF population. **(C)** Venn diagram illustrating the number of overlapping DEGs upregulated in OFs between scRNA-seq and bulk RNA-seq. **(D)** Relative mRNA expression of *BMP4* and *FGF10* in DF and OF groups. **(E)** Immunofluorescence staining and **(F)** MFI of BMP4 in two groups. MFI, Mean Fluorescence Intensity. **(G)** Western blotting and **(H)** quantitative analysis of BMP4 in two groups. **(I)** ELISA of BMP4 in the supernatants of cell cultures from two groups. Scale bars, 100 μm. The results presented are the mean ± SD of six biologically independent replicates. *p < 0.05, **p < 0.01, ***p < 0.001.

**FIGURE 4:**
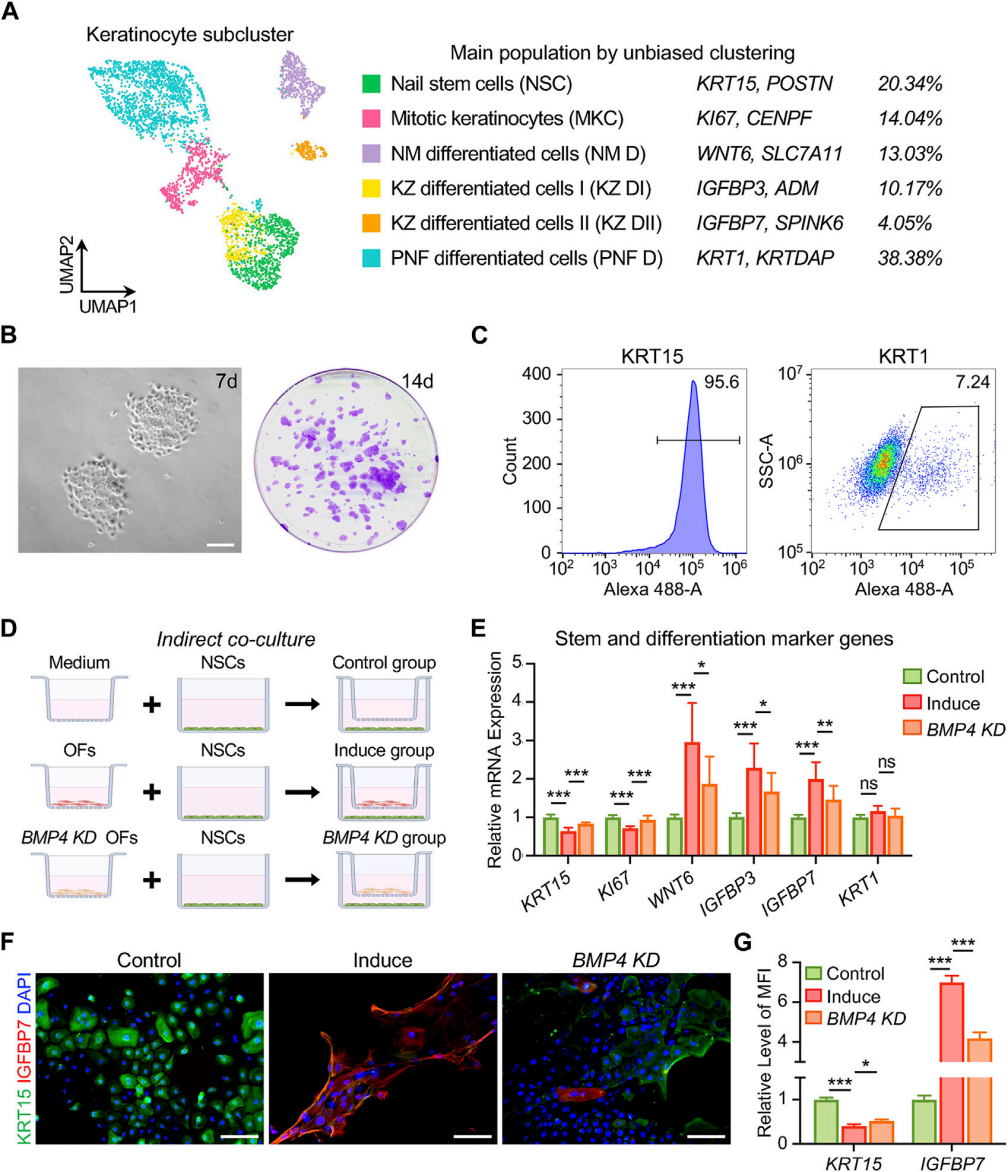
BMP4 derived from human onychofibroblasts regulates differentiation of nail stem cells. **(A)** Identity, marker genes, and cell proportions of human nail keratinocyte subtypes. **(B)** *In vitro* culture showing cell expansion and colony formation of human NSCs. **(C)** Flow cytometric analysis of KRT15 and KRT1 expression in human NSCs cultured *in vitro*. **(D)** Grouping of indirect co-cultures of OFs and NSCs. **(E)** Relative mRNA expression of stem and differentiation marker genes in keratinocytes under different co-culture conditions. **(F)** Immunofluorescence staining and **(G)** MFI of KRT15 and IGFBP7 in three groups. MFI, Mean Fluorescence Intensity. *KD*, *knockdown*. Scale bars, 200 μm. The results presented are the mean ± SD of three biologically independent replicates. *p < 0.05, **p < 0.01, ***p < 0.001, ns, no significant.

To gather additional insights, we reanalyzed scRNA-seq data from human nail units (Kim et al., 2021). This analysis yielded 11,621 high-quality single-cell transcriptome profiles, revealing 8 cell types, including *COL1A1* ^+^ *APOD* ^+^ fibroblasts (Figure 3A and Supplementary Figure S4A–C). We performed a second round of unsupervised clustering on all fibroblasts and found that they could be divided into 12 subpopulations, thus confirming the significant heterogeneity within this cell class (Supplementary Figure S4D). We observed that 5 cell subpopulations (pop. 0, 4, 7, 8, 10) expressed a large number of genes characteristic of human OFs, such as *CD10*, *RSPO4*, *WIF1* and others (Kim et al., 2021; Shim et al., 2022) (Supplementary Figure S4E). We conducted an analysis of DEGs in OFs and non-OF cells, comparing the results with our bulk RNA-seq data (Figures 3B,C). *BMP4* and *FGF10* surfaced as potential soluble mediators for OFs to exert biological functions (Figure 3C). RT-qPCR results confirmed the elevated expression of these genes in OFs (Figure 3D). We prioritized BMP4 due to its association with postnatal digit tip regeneration in mice (Han et al., 2008), which suggests its potential to effectively modulate human NSC differentiation. Immunofluorescence staining, western blotting, and ELISA results collectively demonstrated higher BMP4 protein synthesis and secretion by OFs compared to DFs (Figures 3E–I). Furthermore, scRNA-seq revealed six subtypes of keratinocytes in the human nail epithelium (Supplementary Figure S5A). Alongside KRT15^+^ NSCs and KI67^+^ mitotic cells, WNT6^+^ nail matrix cells (NM D) (Kim et al., 2021), as well as IGFBP3^+^/IGFBP7^+^ cells in the keratogenous zone (KZ DI, KZ DII) and KRT1^+^ cells in the proximal nail fold (PNF D), were identified (Figure 4A, Supplementary Figure S5B,C). Human NSCs were isolated via type IV collagen adherence and cultured in keratinocyte expansion medium. Colony-forming assays and flow cytometric analysis confirmed the maintenance of stemness *in vitro* by KRT15^+^ NSCs (Figures 4B,C). We investigated the impact of OFs-derived BMP4 on human NSC differentiation using an indirect co-culture model (Figure 4D). RT-qPCR analysis revealed that OFs downregulated *KRT15* and *KI67* expression while upregulating *WNT6*, *IGFBP3*, and *IGFBP7* expression through soluble mediator secretion, with no significant change observed in *KRT1* expression (Figure 4E). Knockdown of *BMP4* inhibited these gene expression changes (Figure 4E), indicating that BMP4 from OFs induces NSC differentiation into nail matrix and keratogenous zone cells. Immunofluorescence staining of KRT15 and IGFBP7 corroborated these findings (Figures 4F,G). Thus, BMP4 emerges as an important signal for human onychofibroblasts in regulating NSC differentiation, shedding light on the nail microenvironment and NSC differentiation mechanisms.

### BMP4 induces *in vitro* differentiation of human NSCs through the TGF-beta signaling pathway

**FIGURE 5:**
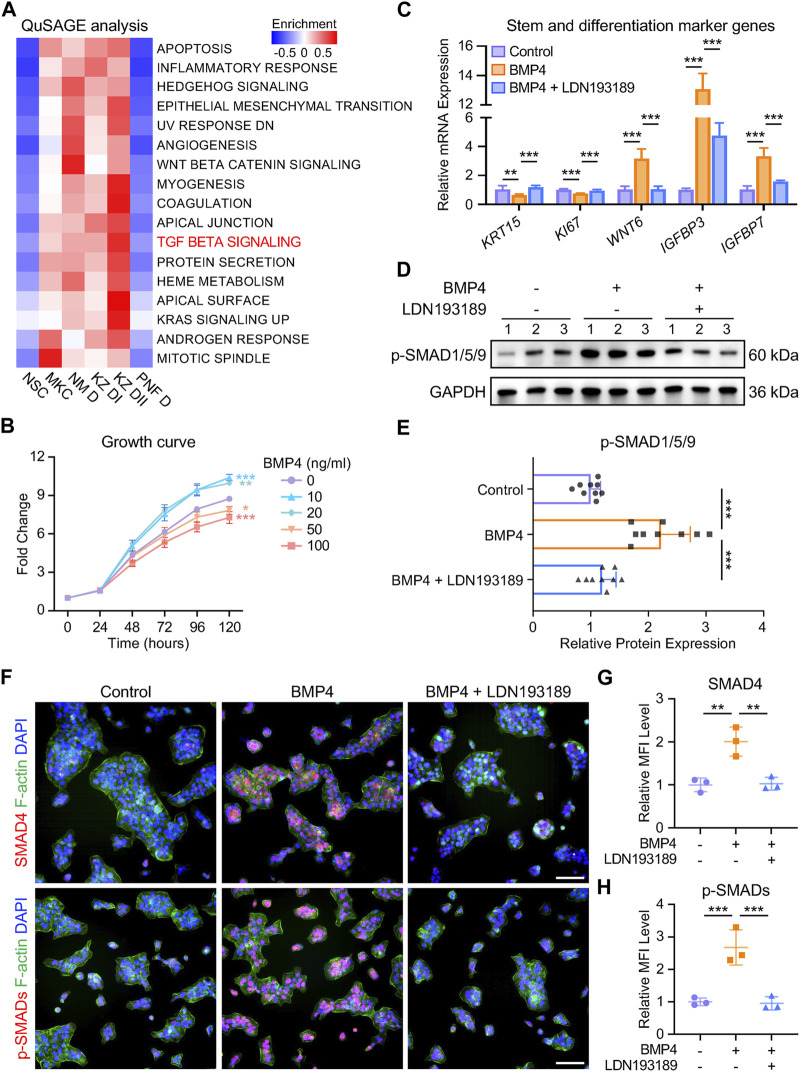
BMP4 induces *in vitro* differentiation of human NSCs through the TGF-beta signaling pathway. **(A)** QuSAGE analysis of human nail keratinocyte populations. **(B)** Effect of recombinant human BMP4 on NSC proliferation. **(C)** Relative mRNA expression of stem and differentiation marker genes in keratinocytes under different treatments. **(D)** Western blotting and **(E)** quantitative analysis of p-SMADs in three groups. The results presented are the mean ± SD of nine biologically independent replicates. **(F)** Immunofluorescence staining and **(G, H)** MFI of SMAD4 and p-SMADs in three groups. MFI, Mean Fluorescence Intensity. Scale bars, 100 μm. Unless otherwise stated, the results presented are the mean ± SD of three biologically independent replicates. *p < 0.05, **p < 0.01, ***p < 0.001.

To investigate the downstream signaling pathways activated by BMP4, we performed QuSAGE analysis on the nail keratinocyte populations. We identified 17 significantly enriched gene sets, including the TGF-beta signaling pathway, in NM D, KZ DI, and KZ DII subtypes (Figure 5A). While TGF-beta signaling is known to be crucial for both stem cell maintenance and differentiation (Watabe and Miyazono, 2009), its role in human NSCs required confirmation. We treated NSCs with recombinant human BMP4 protein and found that a high concentration of 50 ng/mL prompted NSCs to exit the cell cycle and commit to differentiation (Figure 5B). RT-qPCR analysis revealed a decrease in the expression of the stem cell marker *KRT15* and a significant increase in the expression of differentiated cell marker genes in the BMP4-treated group compared to the control group, with attenuation of these effects observed upon co-treatment with the BMP type I receptor inhibitor LDN193189 (0.5 μM) (Figure 5C). We then examined the protein levels of SMAD effectors, the critical components of the TGF-beta signaling pathway. We found increased phosphorylation of SMAD1/5/9 in the BMP4-treated group by Western blotting, suggesting the activation of the TGF-beta signaling pathway (Figures 5D,E). Co-treatment with the BMP receptor inhibitor reduced the effect of BMP4 on p-SMAD1/5/9 (p-SMADs) protein expression in NSCs (Figures 5D,E). Immunofluorescence assays examining the intracellular distribution of p-SMADs and SMAD4 revealed enhanced nuclear accumulation of SMAD1/5/9/4 complexes after BMP4 treatment, whereas LDN193189 notably decreased nuclear accumulation of the complexes (Figures 5F–H). These findings demonstrate that BMP4 drives *in vitro* differentiation of human NSCs by binding to BMP receptors and activating the TGF-beta signaling pathway. The elucidation of this molecular mechanism is critical for understanding mesenchymal-epithelial interactions and stem cell differentiation events in the human nail unit.

## Discussion

Fibroblasts play crucial roles in development/growth, homeostasis, and the injury response; they are indispensable in forming and maintaining the structure of virtually every organ and are key contributors to the tissue repair process (Plikus et al., 2021; Talbott et al., 2022). Recent studies have highlighted the remarkable heterogeneity among fibroblasts, with significant phenotypic and functional diversity observed between and within tissues (Lynch and Watt, 2018; Talbott et al., 2022). In the context of human nails, onychofibroblasts (OFs) have been proposed to exhibit distinct gene expression patterns compared to surrounding dermal fibroblasts (Lee et al., 2006; Lee et al., 2012; Park et al., 2017). However, the specific cellular properties of OFs and their role in regulating nail stem cells (NSCs) remain poorly understood.

Our study provides several key insights. Firstly, we demonstrate that OFs exhibit mesenchymal stem cell (MSC) -like characteristics, challenging the traditional view of fibroblasts as terminally differentiated cells. Our findings indicate that OFs express MSC markers and possess multilineage differentiation potential, particularly towards adipogenic, osteogenic, and chondrogenic lineages. Additionally, we observed heightened expression of key transcription factors associated with dermal stem cells in OFs, including *TWIST1, MSX1,* and *MSX2* (Thulabandu et al., 2018) (Supplementary Figure S6). The stem cell properties exhibited by OFs may correlate with their involvement in guiding nail development. Future investigations are warranted to clarify the *in vivo* differentiation and self-renewal potential of human OFs.

Moreover, similar to specialized dermal papilla cells in hair follicles (Chen et al., 2020; Liu et al., 2022), we identified OFs as the principal signaling niche for nail epithelial cells. OFs exhibit distinctive transcriptional profiles and actively participate in mesenchymal-epithelial signaling communication, involving various signaling pathways such as TGF-beta, Wnt, and Notch. Importantly, we propose that OFs regulate NSC differentiation via the BMP4 signal.

BMP4, a member of the secreted bone morphogenetic protein family and a component of the TGFβ superfamily, binds to and activates type I and type II serine/threonine kinase receptors, initiating downstream signaling through both SMAD-dependent and SMAD-independent pathways (Bragdon et al., 2011; Derynck and Zhang, 2003). It has several conserved roles in body patterning and morphogenesis, including limb and digit formation, tooth development, cartilage and bone induction, and the development of various tissue organs (Blackburn et al., 2019).

In our study, we observed that OFs synthesize and secrete more BMP4 protein. BMP4 subsequently triggers the phosphorylation of SMAD1/5/9 in NSCs by binding to BMP type I receptors, facilitating the translocation of the SMAD4 complex into the nucleus, and thereby enhancing the transcription of differentiation genes. It is notable that only the marker genes of nail matrix and keratogenous zone cells upregulated, while no significant change was detected in the expression of the peri-nail epidermal cell marker *KRT1*. Interestingly, Leung et al. demonstrated that Bmpr1a-mediated signaling is crucial for proper nail differentiation in mice and without it, nail differentiation is compromised and adopts an epidermal fate (Leung et al., 2014), which aligns closely with our findings. Overall, our study elucidates the specific signaling mechanism underlying NSC differentiation mediated by BMP4 and reveals the downstream pathway activation during this process.

**FIGURE 6:**
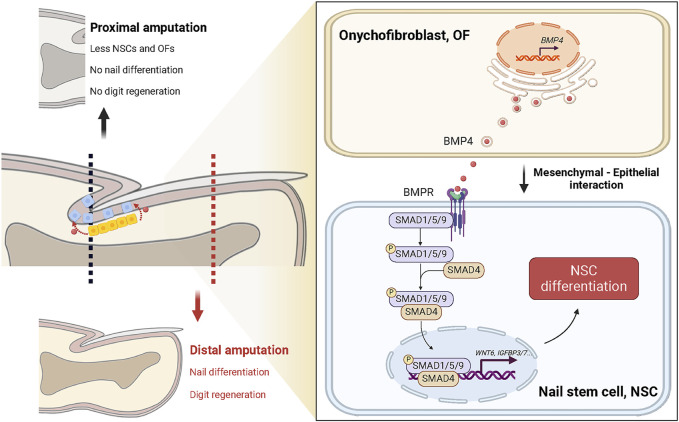
Proposed model: Onychofibroblast-mediated nail stem cell differentiation links amputation level with digit regeneration capacity. Under homeostatic conditions, BMP4 secreted by onychofibroblasts (OFs) activates the TGF-beta signaling pathway in nail stem cells (NSCs) and induces cell differentiation. It is known that NSCs and the mechanisms governing NSC differentiation are directly related to their ability to orchestrate digit regeneration. In instances of distal amputation, NSCs and OFs remain largely unaffected; regenerating nail epithelial cells can cover the wound site, preserving nail differentiation, and facilitating complete digit regeneration. Conversely, proximal amputation leads to decreased NSCs and OFs, along with diminished differentiation signals, resulting in impaired nail and digit regeneration.

Furthermore, we explore the potential connection between OFs, NSCs, and digit regeneration (Figure 6). Takeo et al. emphasized that NSC differentiation signals couple nail growth to digit regeneration (Takeo et al., 2013). Previous research has demonstrated that distal amputation ensures successful digit regeneration, while proximal amputation removes the visible nail plate, over 50% of the distal phalanx, and the nail matrix’s distal region, resulting in complete regeneration failure. In the latter cases, NSCs in the proximal matrix and residual OFs are significantly reduced, leading to attenuation of differentiation signals and defective nail differentiation. Although our study did not directly assess the impact of OF-derived BMP4 on digit regeneration, evidence from the research of Han et al. suggests a potential link. They found that digits of *Msx1*-deprived mice exhibited downregulated expression of *Bmp4* and displayed regeneration defects, which were reversed after administration of exogenous Bmp4 (Han et al., 2003).

In summary, our data showed the mesenchymal stem cell-like properties and unique transcriptional profiles of human OFs. We elucidated the mechanism by which OFs regulate NSC differentiation towards a nail epithelial fate through BMP4 signaling. These findings underscore the importance of the specialized dermal microenvironment in shaping NSCs’ phenotype and maintaining nail homeostasis. However, it is imperative to acknowledge that further investigation is required to elucidate the *in vivo* effect of NSC differentiation mediated by the BMP4/BMPR/SMADs signaling pathway on digit regeneration. From a translational point of view, exploring NSCs and their intricate interactions with surrounding tissues like mesenchyme, periosteum, innervation, and vasculature offers a unique platform from which novel therapies could develop for the treatment of nail and digit defects and, in a broader sense, therapies for patients with severe limb amputation.

## Data Availability

The datasets presented in this study can be found in online repositories. The names of the repository/repositories and accession number(s) can be found below: https://www.ncbi.nlm.nih.gov/geo/query/acc.cgi?acc=GSE263876.
