# Supplementary figures and images for: Integrating scRNA-seq and bulk RNA-seq to explore the differentiation mechanism of human nail stem cells mediated by onychofibroblasts
Source: Front Cell Dev Biol. 2024 Jun 3;12:1416780. doi: 10.3389/fcell.2024.1416780 (PMC11181305; doi:10.3389/fcell.2024.1416780)

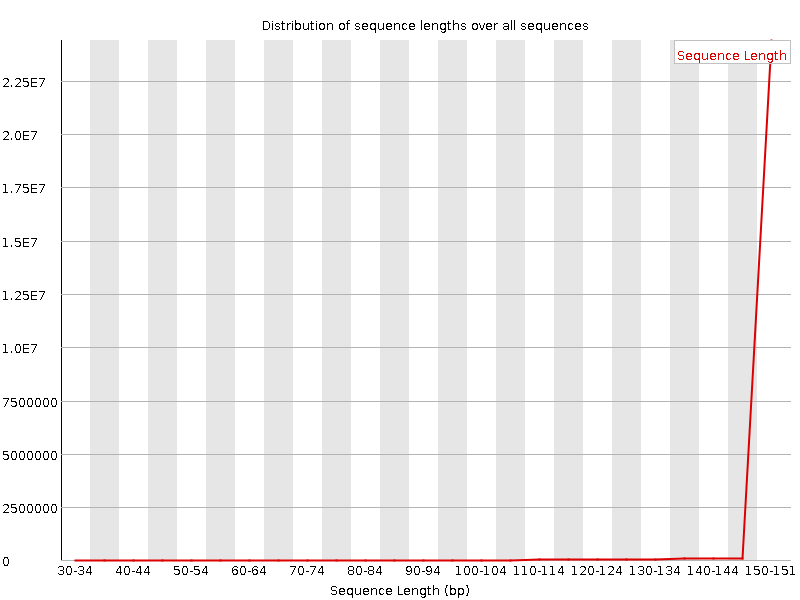

Supplement: Supplementary file 1 [file DataSheet1.ZIP › quality control/OF3_1/sequence_length_distribution.png]

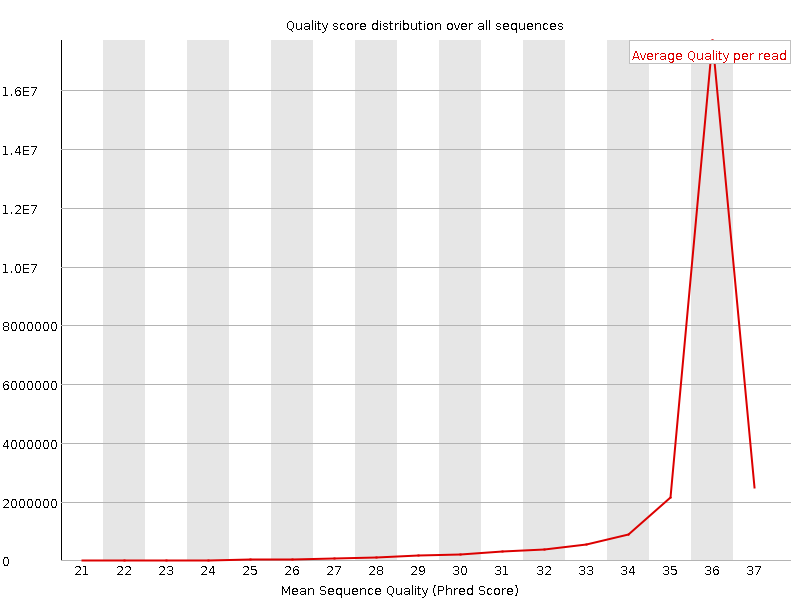

Supplement: Supplementary file 1 [file DataSheet1.ZIP › quality control/OF3_1/per_sequence_quality.png]

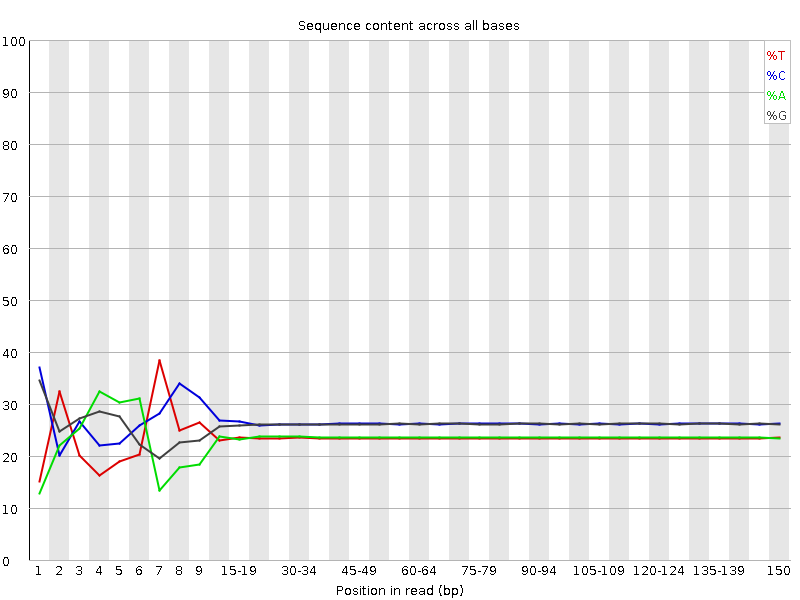

Supplement: Supplementary file 1 [file DataSheet1.ZIP › quality control/OF3_1/per_base_sequence_content.png]

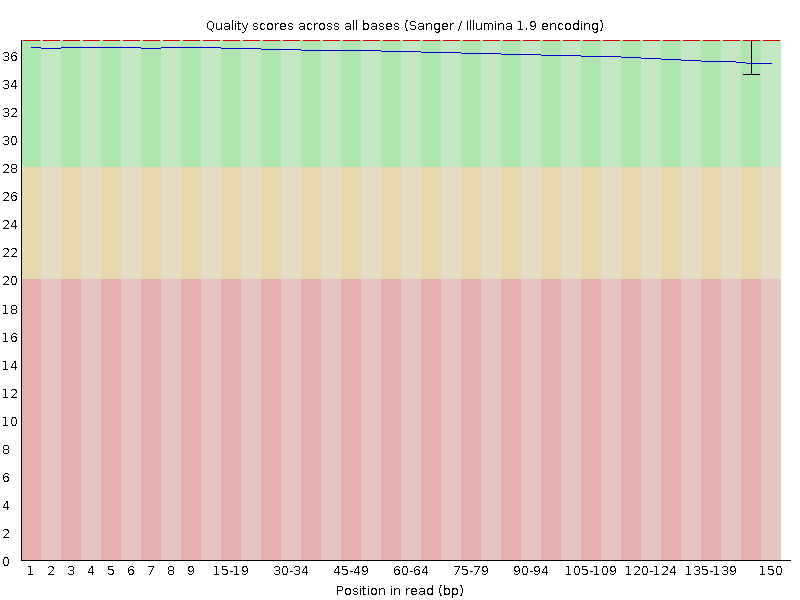

Supplement: Supplementary file 1 [file DataSheet1.ZIP › quality control/OF3_1/per_base_quality.png]

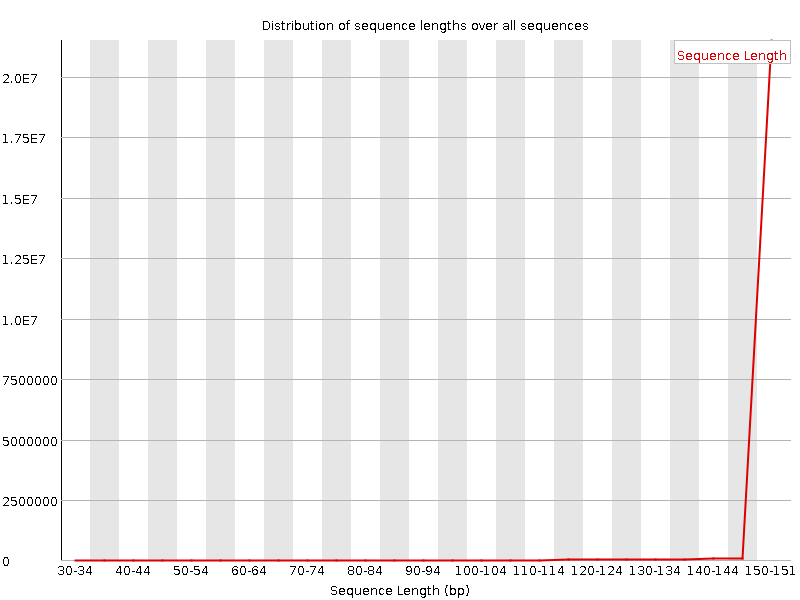

Supplement: Supplementary file 1 [file DataSheet1.ZIP › quality control/OF1_2/sequence_length_distribution.png]

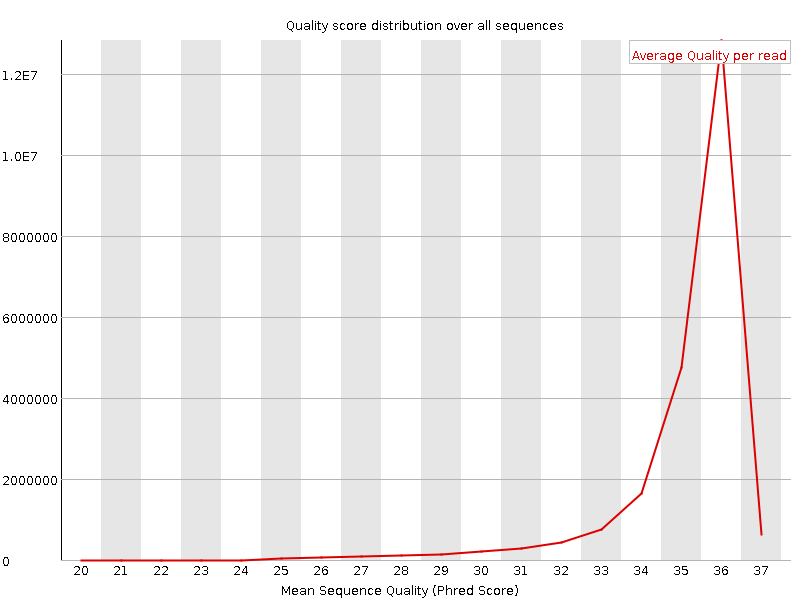

Supplement: Supplementary file 1 [file DataSheet1.ZIP › quality control/OF1_2/per_sequence_quality.png]

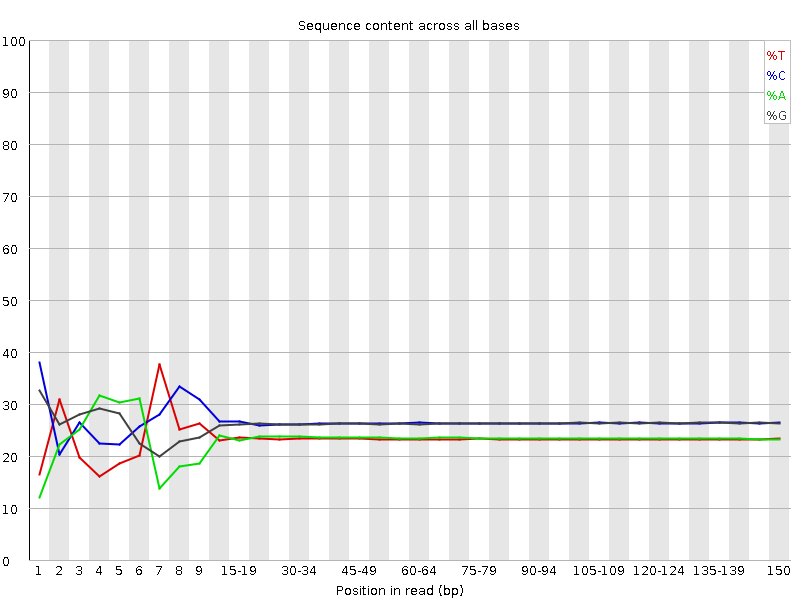

Supplement: Supplementary file 1 [file DataSheet1.ZIP › quality control/OF1_2/per_base_sequence_content.png]

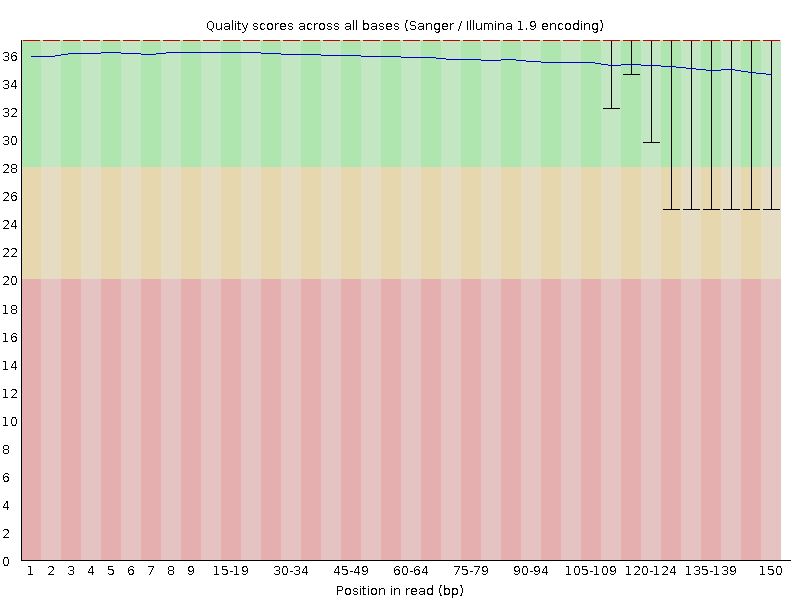

Supplement: Supplementary file 1 [file DataSheet1.ZIP › quality control/OF1_2/per_base_quality.png]

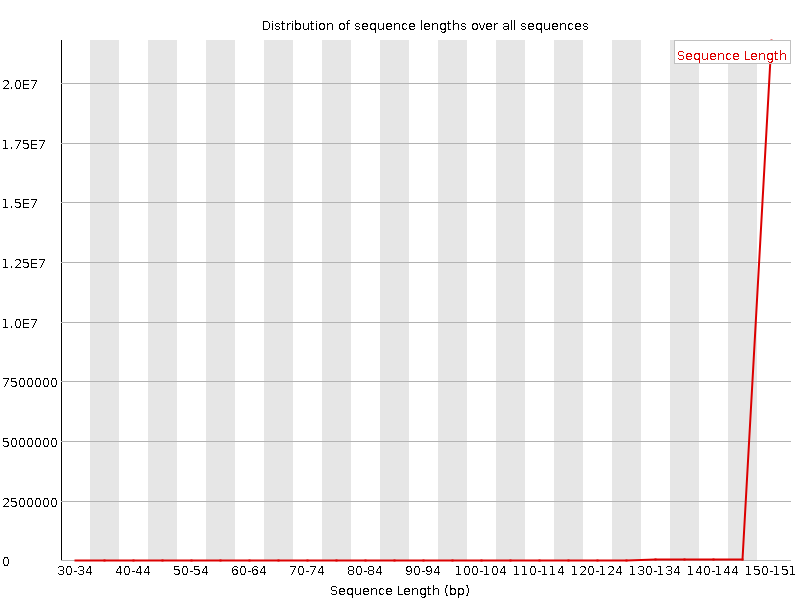

Supplement: Supplementary file 1 [file DataSheet1.ZIP › quality control/DF2_1/sequence_length_distribution.png]

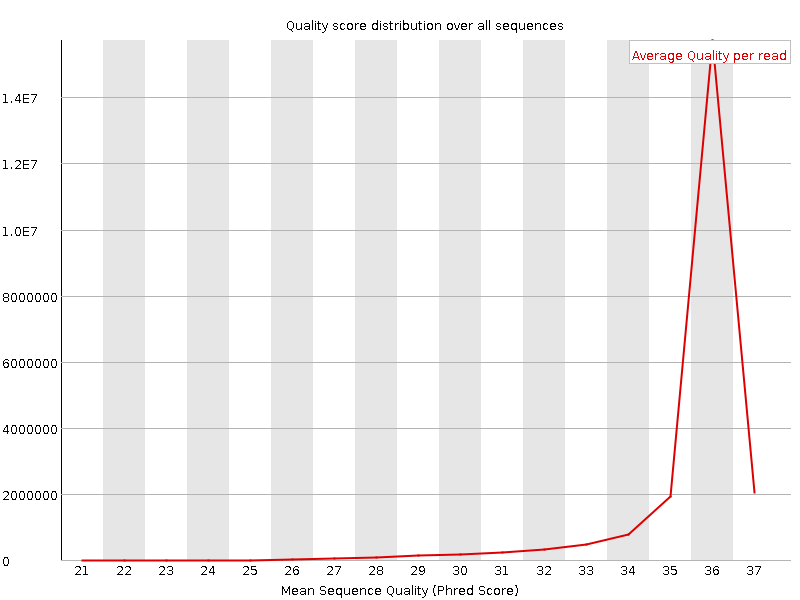

Supplement: Supplementary file 1 [file DataSheet1.ZIP › quality control/DF2_1/per_sequence_quality.png]

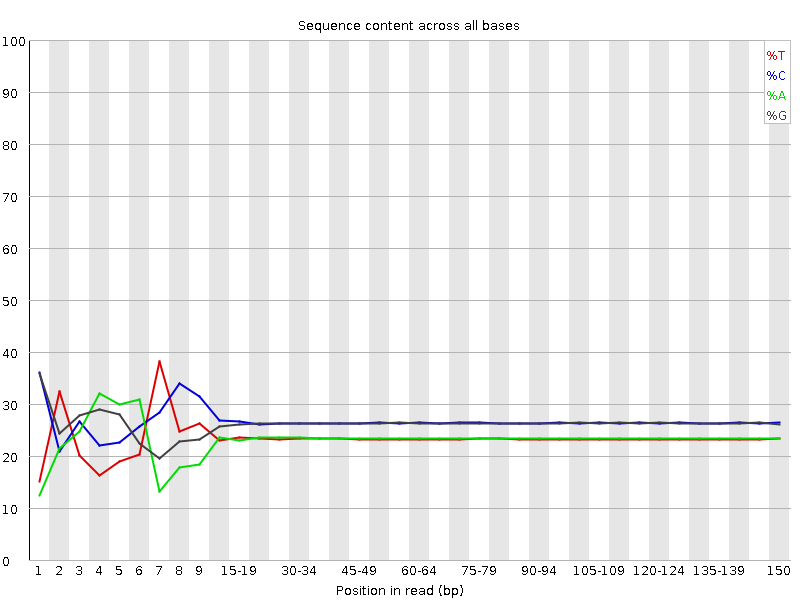

Supplement: Supplementary file 1 [file DataSheet1.ZIP › quality control/DF2_1/per_base_sequence_content.png]

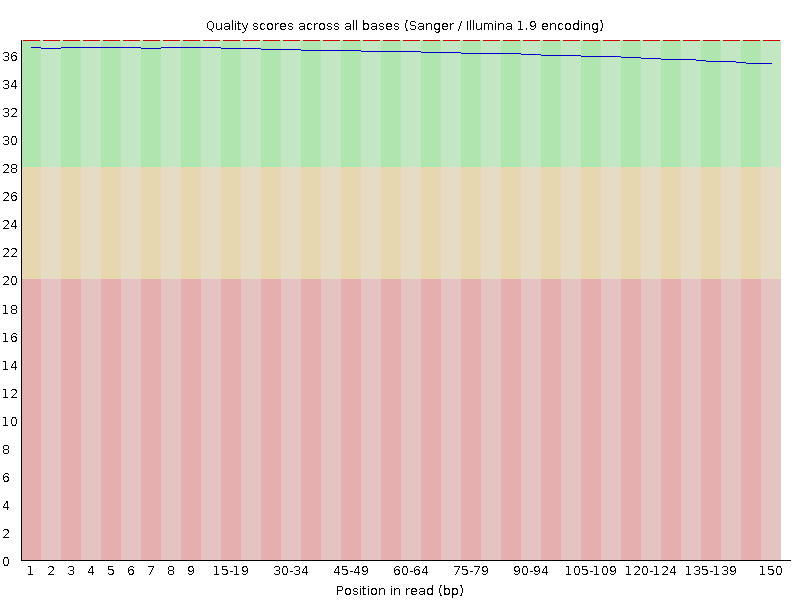

Supplement: Supplementary file 1 [file DataSheet1.ZIP › quality control/DF2_1/per_base_quality.png]

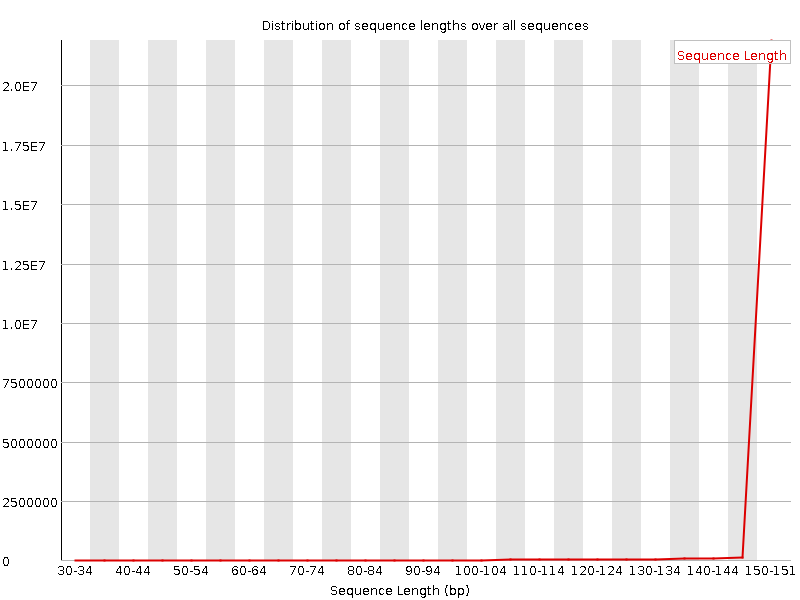

Supplement: Supplementary file 1 [file DataSheet1.ZIP › quality control/OF2_2/sequence_length_distribution.png]

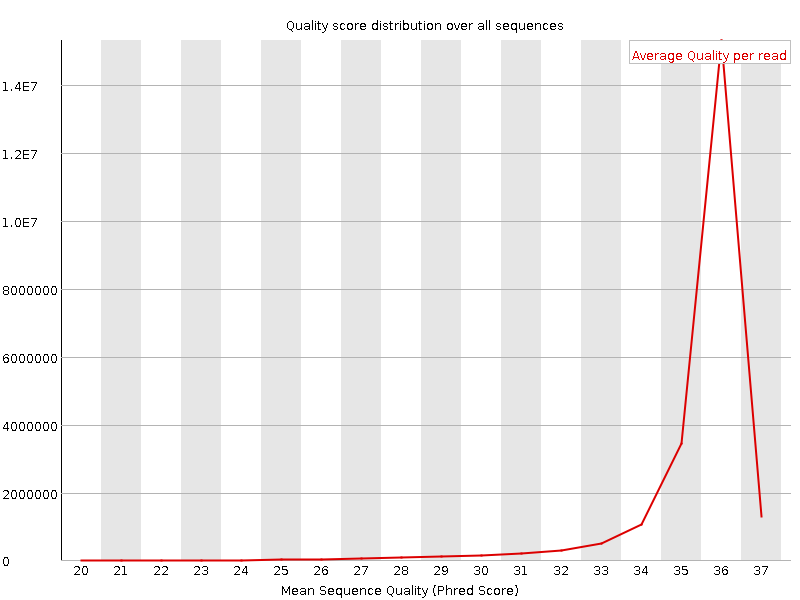

Supplement: Supplementary file 1 [file DataSheet1.ZIP › quality control/OF2_2/per_sequence_quality.png]

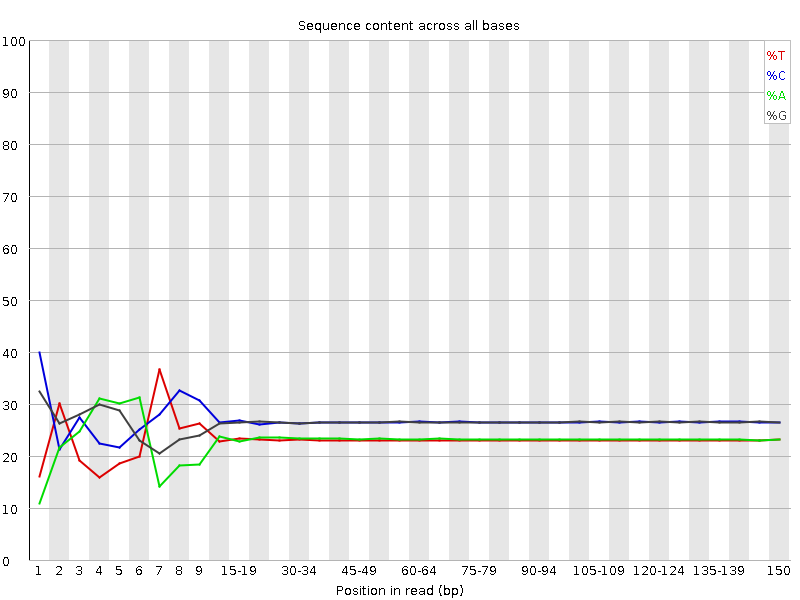

Supplement: Supplementary file 1 [file DataSheet1.ZIP › quality control/OF2_2/per_base_sequence_content.png]

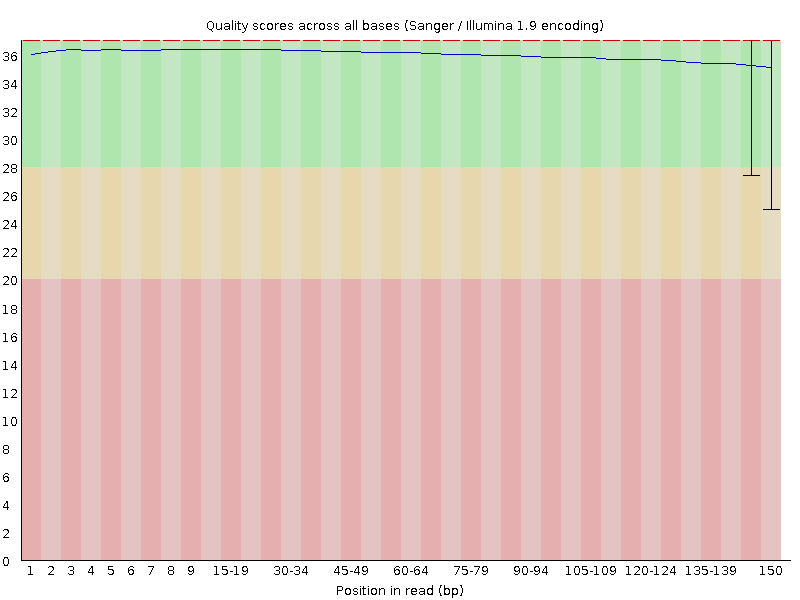

Supplement: Supplementary file 1 [file DataSheet1.ZIP › quality control/OF2_2/per_base_quality.png]

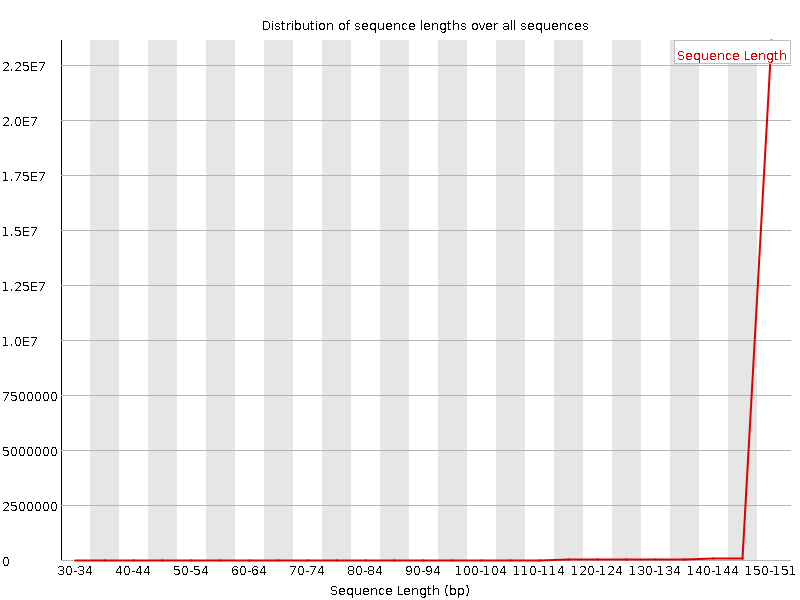

Supplement: Supplementary file 1 [file DataSheet1.ZIP › quality control/DF3_3/sequence_length_distribution.png]

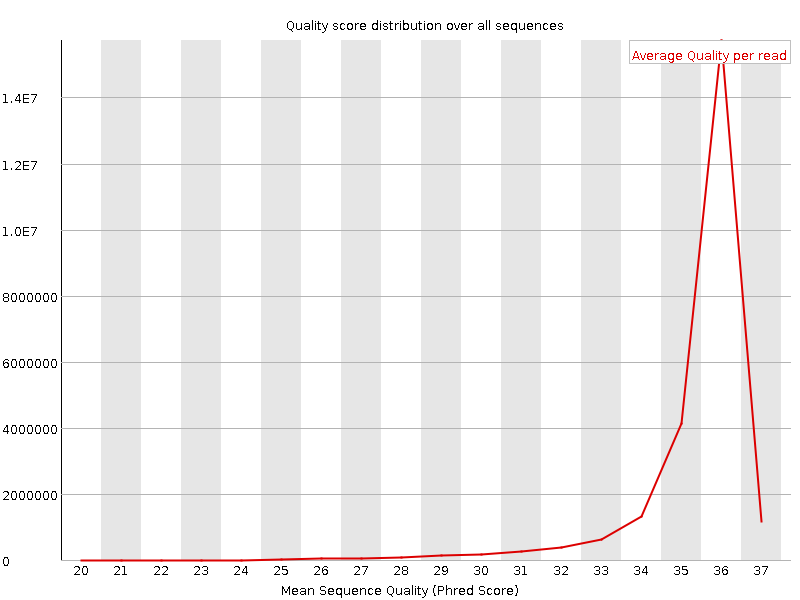

Supplement: Supplementary file 1 [file DataSheet1.ZIP › quality control/DF3_3/per_sequence_quality.png]

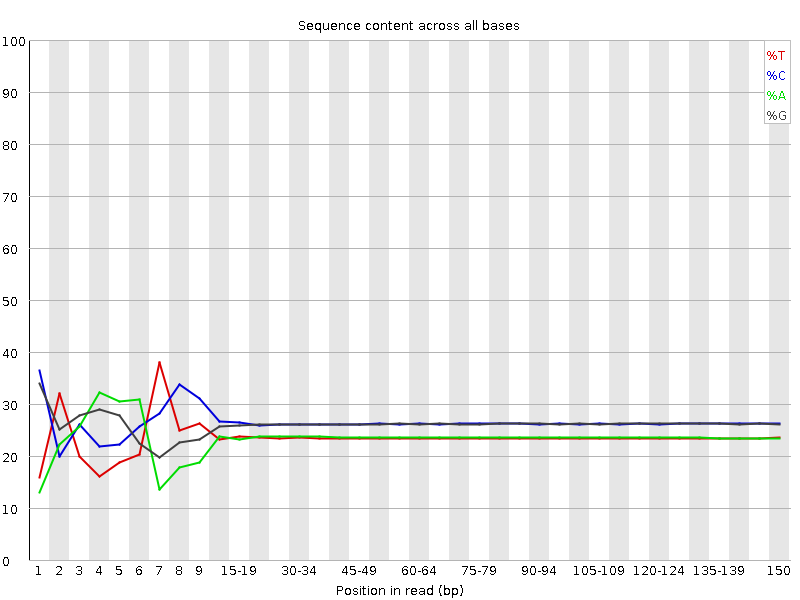

Supplement: Supplementary file 1 [file DataSheet1.ZIP › quality control/DF3_3/per_base_sequence_content.png]

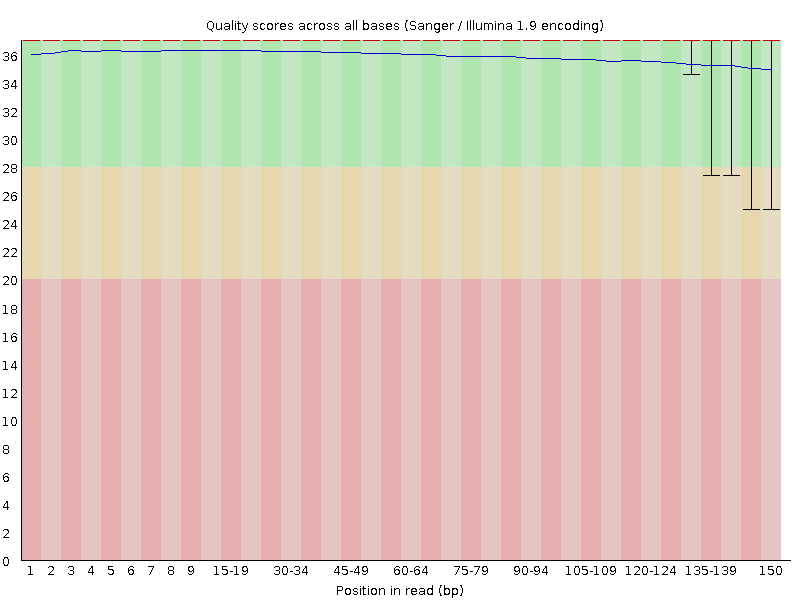

Supplement: Supplementary file 1 [file DataSheet1.ZIP › quality control/DF3_3/per_base_quality.png]

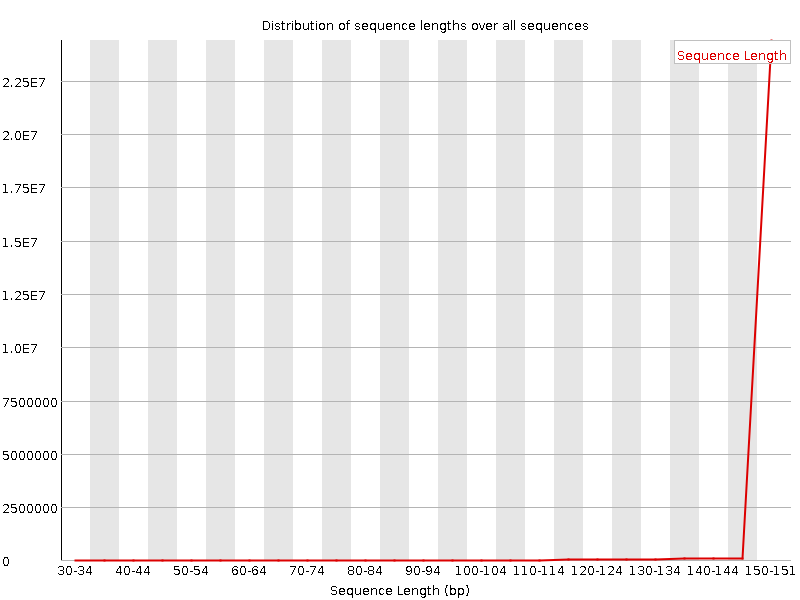

Supplement: Supplementary file 1 [file DataSheet1.ZIP › quality control/DF1_1/sequence_length_distribution.png]

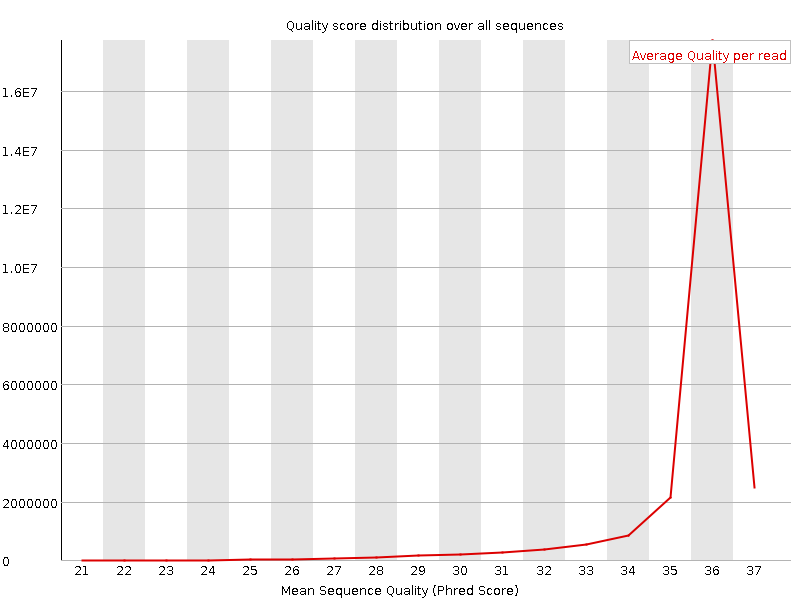

Supplement: Supplementary file 1 [file DataSheet1.ZIP › quality control/DF1_1/per_sequence_quality.png]

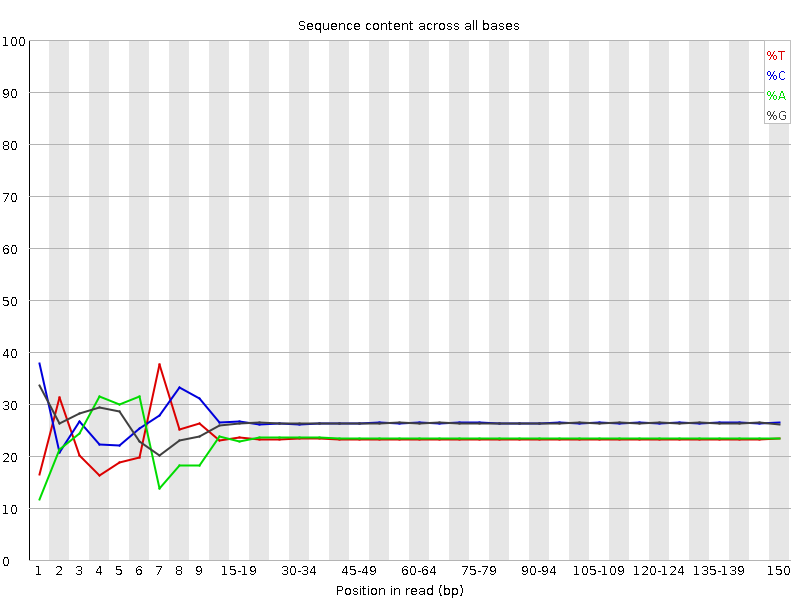

Supplement: Supplementary file 1 [file DataSheet1.ZIP › quality control/DF1_1/per_base_sequence_content.png]

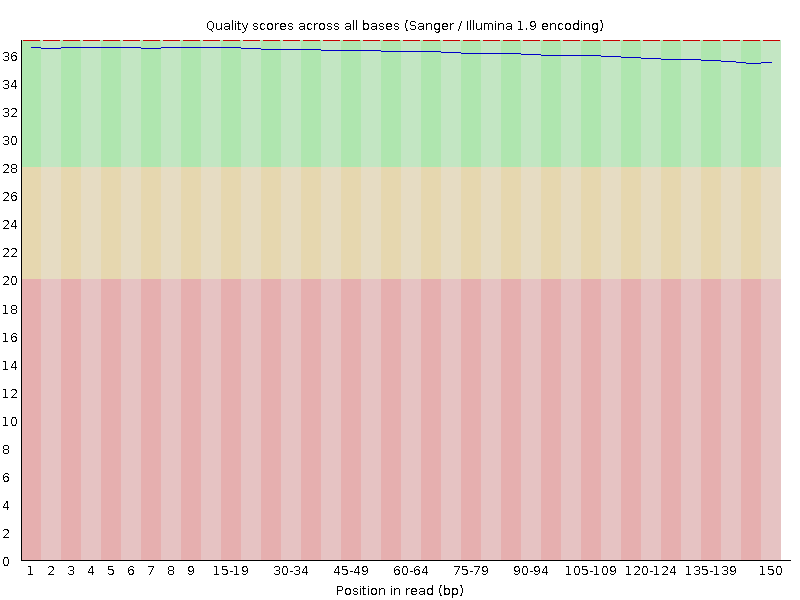

Supplement: Supplementary file 1 [file DataSheet1.ZIP › quality control/DF1_1/per_base_quality.png]

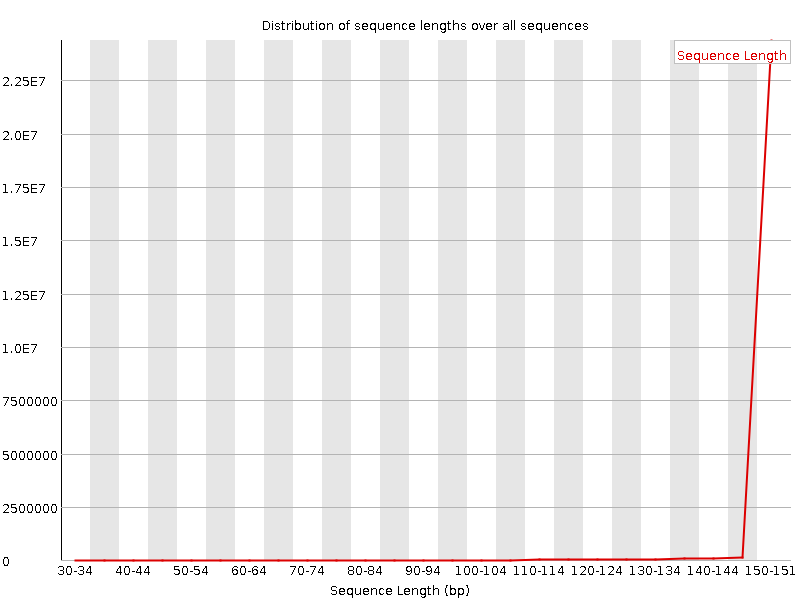

Supplement: Supplementary file 1 [file DataSheet1.ZIP › quality control/OF3_2/sequence_length_distribution.png]

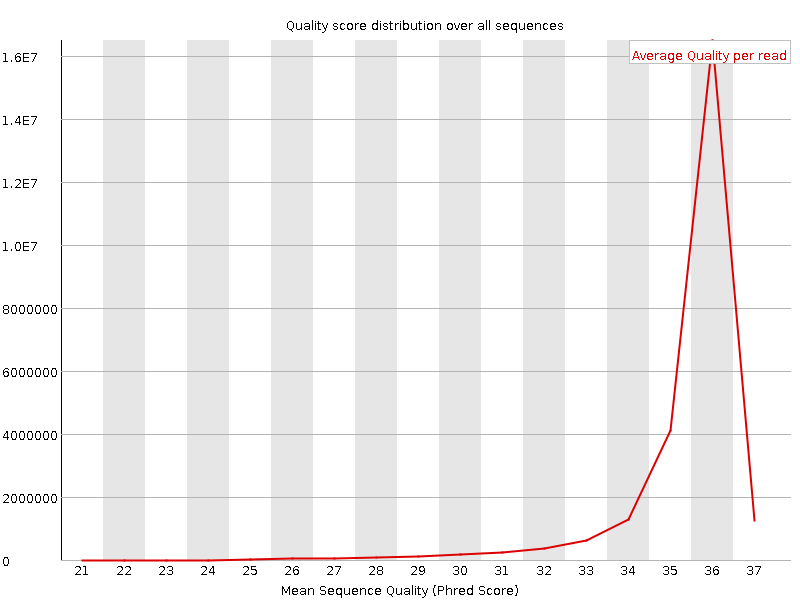

Supplement: Supplementary file 1 [file DataSheet1.ZIP › quality control/OF3_2/per_sequence_quality.png]

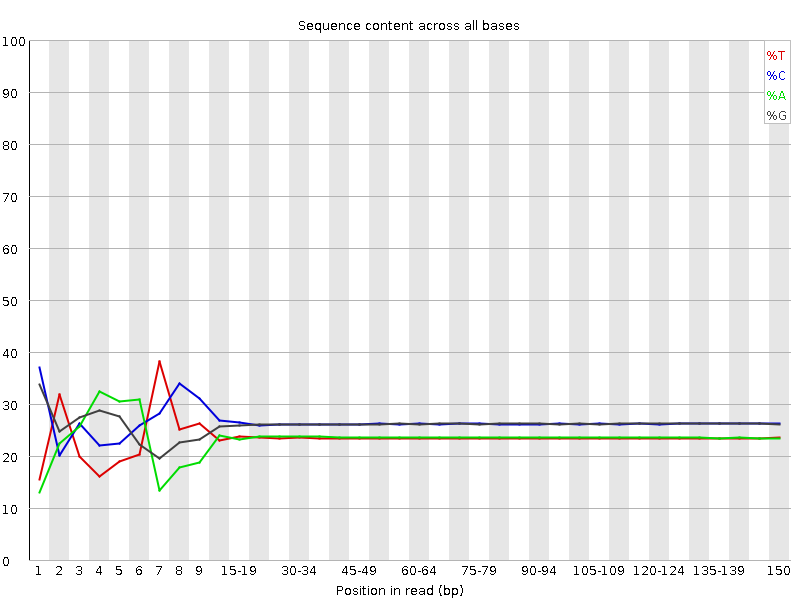

Supplement: Supplementary file 1 [file DataSheet1.ZIP › quality control/OF3_2/per_base_sequence_content.png]

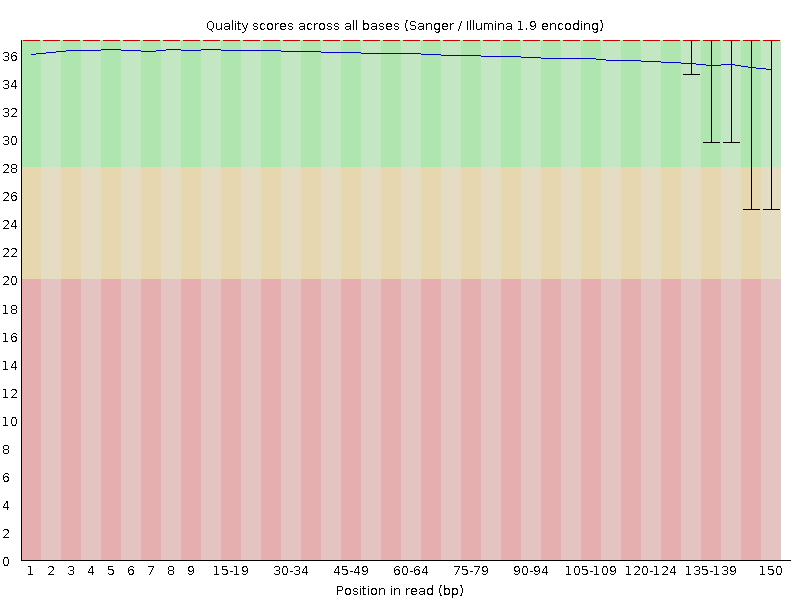

Supplement: Supplementary file 1 [file DataSheet1.ZIP › quality control/OF3_2/per_base_quality.png]

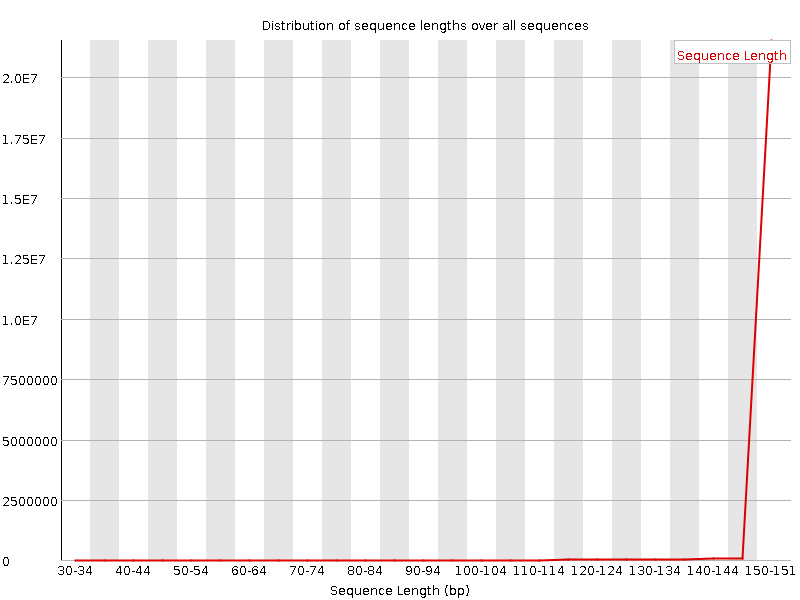

Supplement: Supplementary file 1 [file DataSheet1.ZIP › quality control/OF1_1/sequence_length_distribution.png]

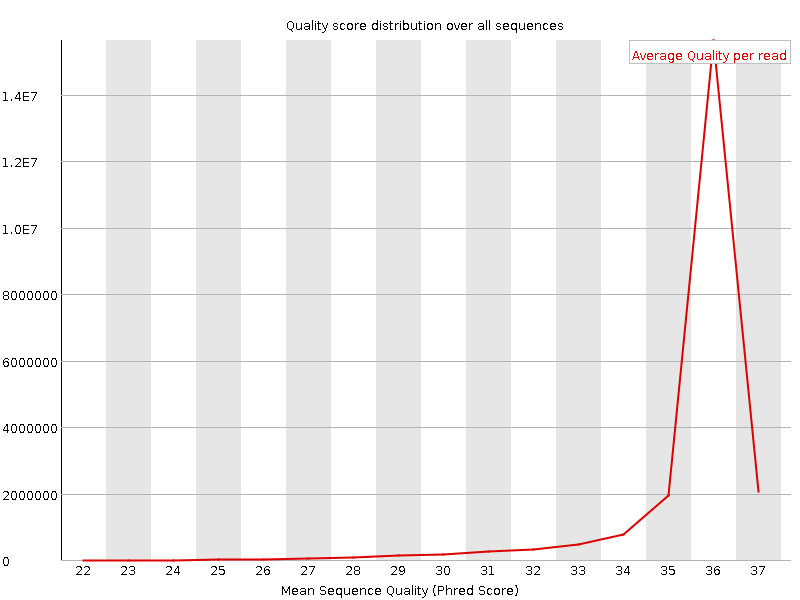

Supplement: Supplementary file 1 [file DataSheet1.ZIP › quality control/OF1_1/per_sequence_quality.png]

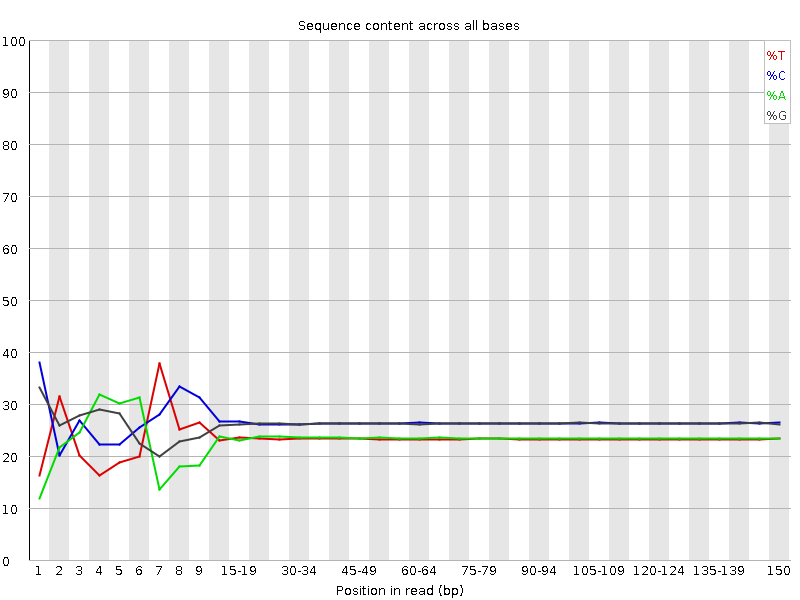

Supplement: Supplementary file 1 [file DataSheet1.ZIP › quality control/OF1_1/per_base_sequence_content.png]

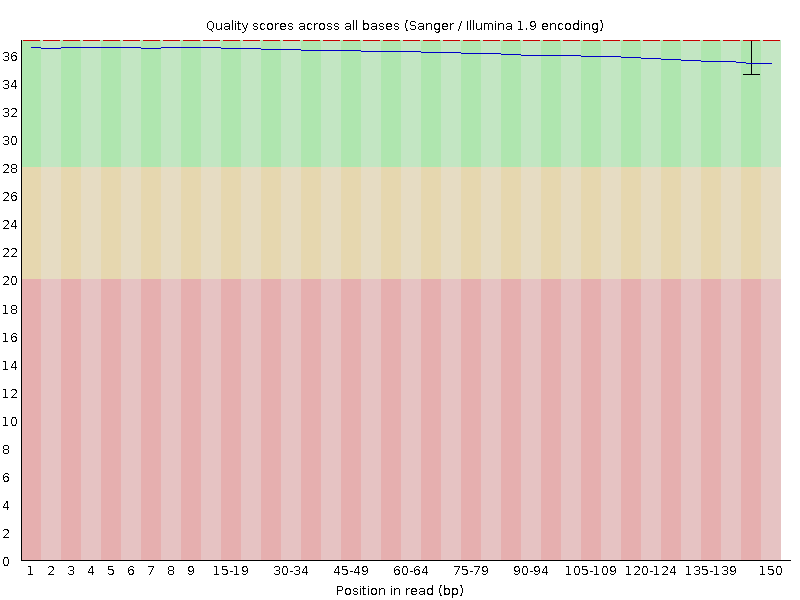

Supplement: Supplementary file 1 [file DataSheet1.ZIP › quality control/OF1_1/per_base_quality.png]

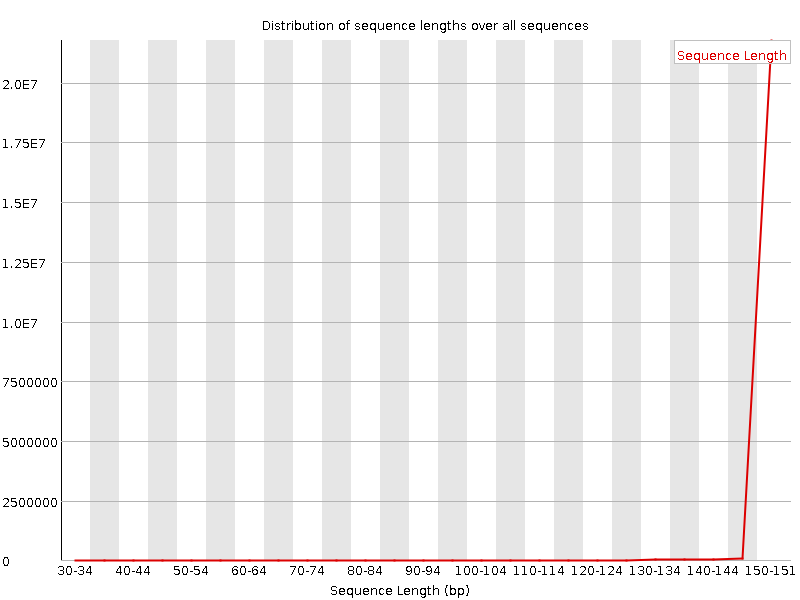

Supplement: Supplementary file 1 [file DataSheet1.ZIP › quality control/DF2_2/sequence_length_distribution.png]

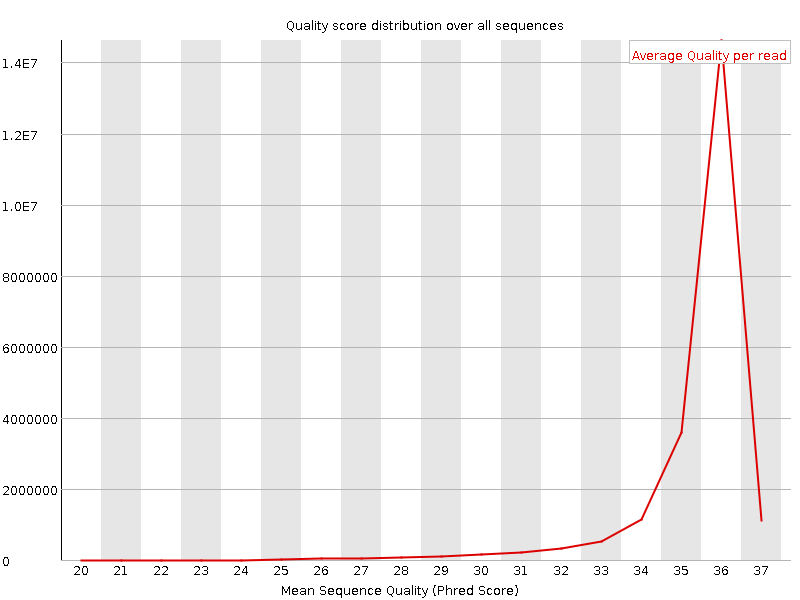

Supplement: Supplementary file 1 [file DataSheet1.ZIP › quality control/DF2_2/per_sequence_quality.png]

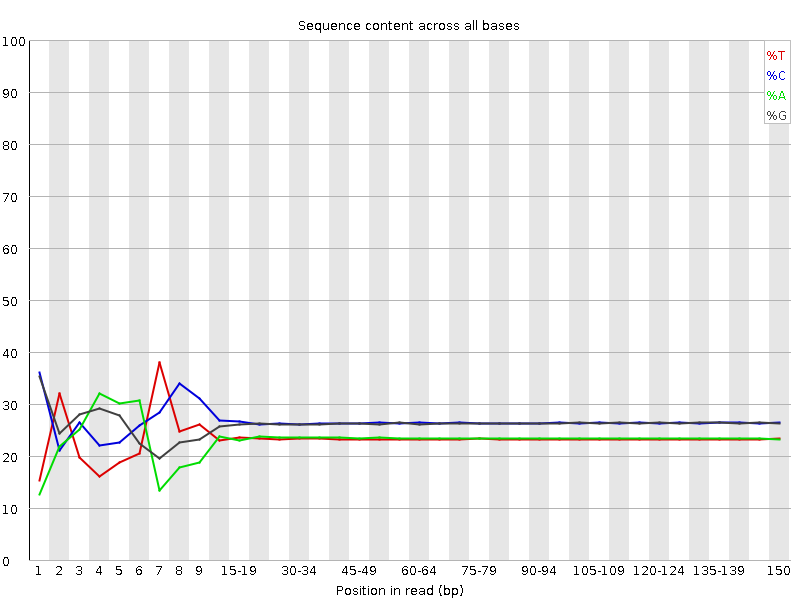

Supplement: Supplementary file 1 [file DataSheet1.ZIP › quality control/DF2_2/per_base_sequence_content.png]

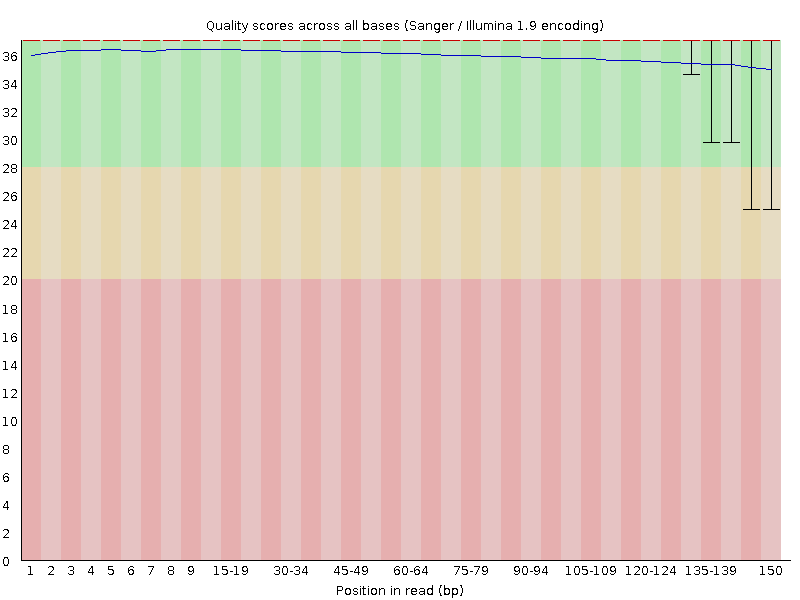

Supplement: Supplementary file 1 [file DataSheet1.ZIP › quality control/DF2_2/per_base_quality.png]

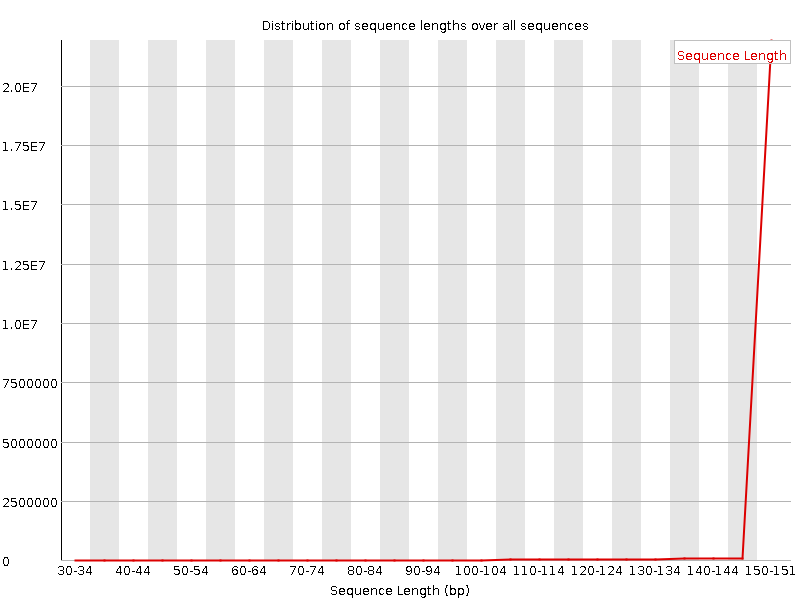

Supplement: Supplementary file 1 [file DataSheet1.ZIP › quality control/OF2_1/sequence_length_distribution.png]

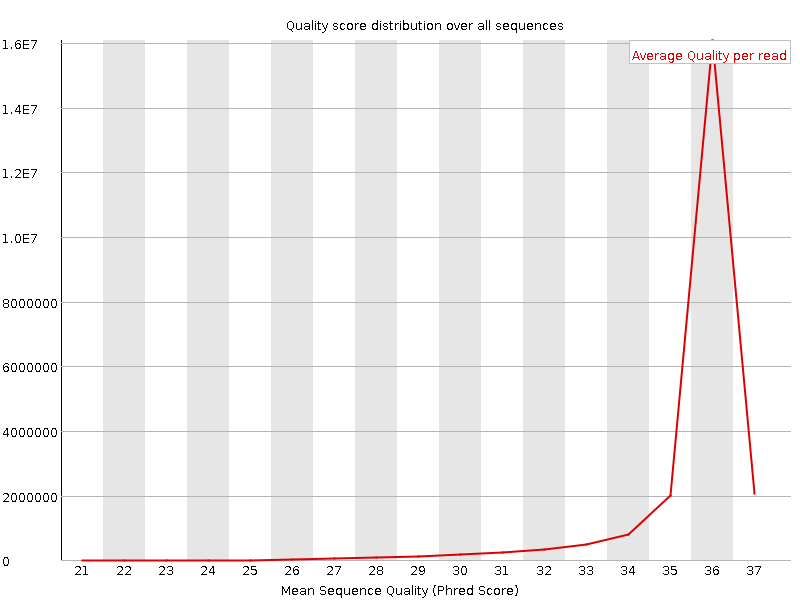

Supplement: Supplementary file 1 [file DataSheet1.ZIP › quality control/OF2_1/per_sequence_quality.png]

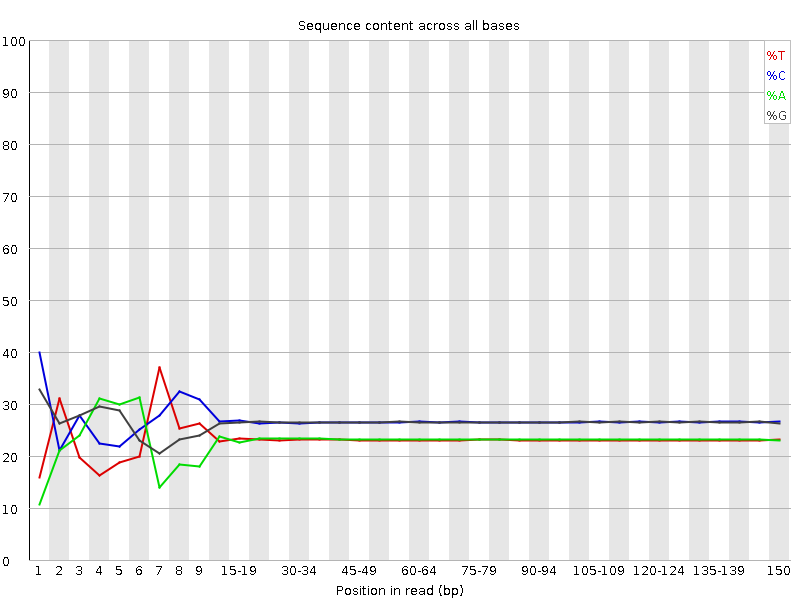

Supplement: Supplementary file 1 [file DataSheet1.ZIP › quality control/OF2_1/per_base_sequence_content.png]

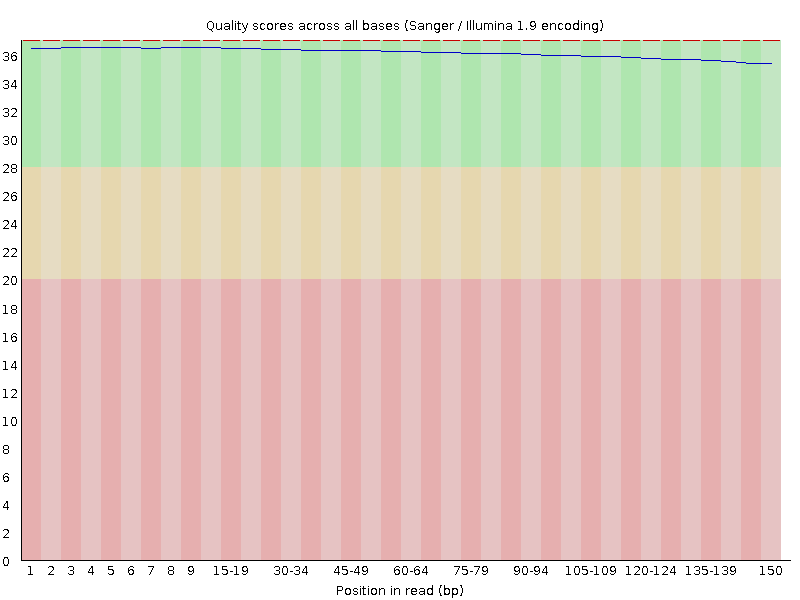

Supplement: Supplementary file 1 [file DataSheet1.ZIP › quality control/OF2_1/per_base_quality.png]

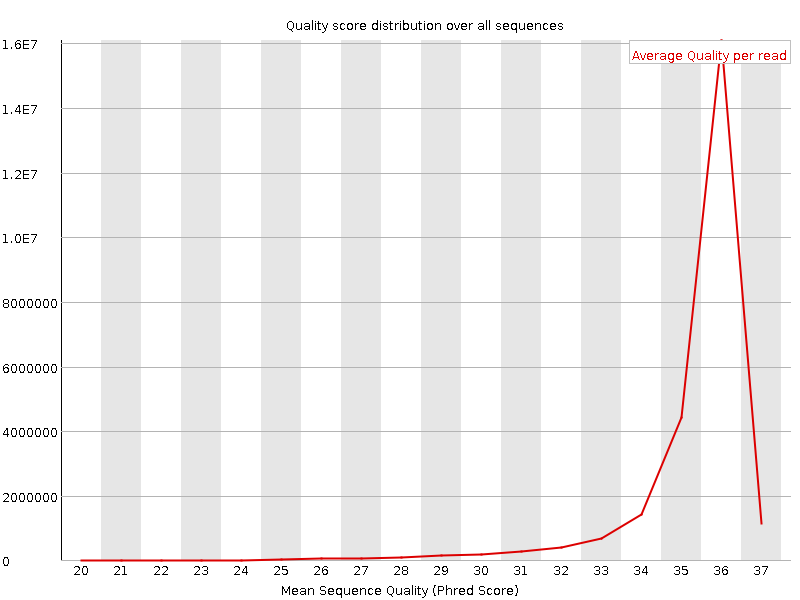

Supplement: Supplementary file 1 [file DataSheet1.ZIP › quality control/DF1_2/per_sequence_quality.png]

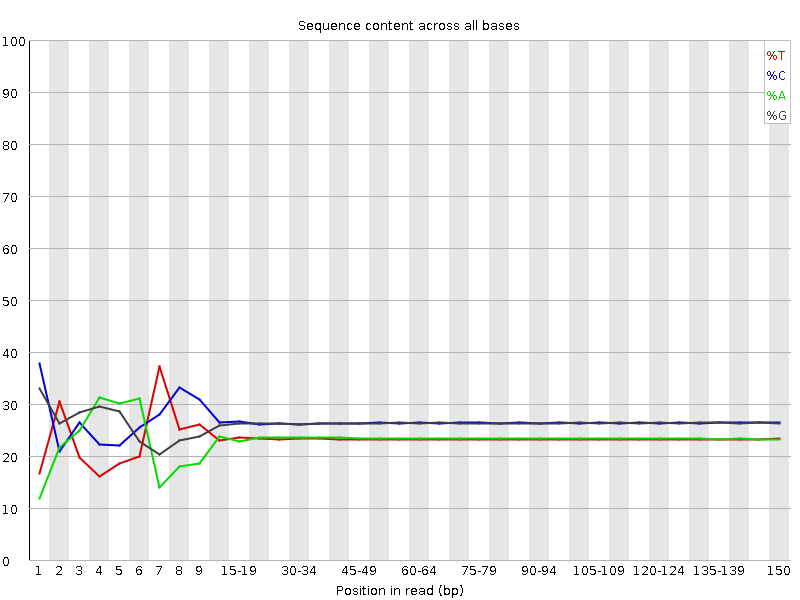

Supplement: Supplementary file 1 [file DataSheet1.ZIP › quality control/DF1_2/per_base_sequence_content.png]

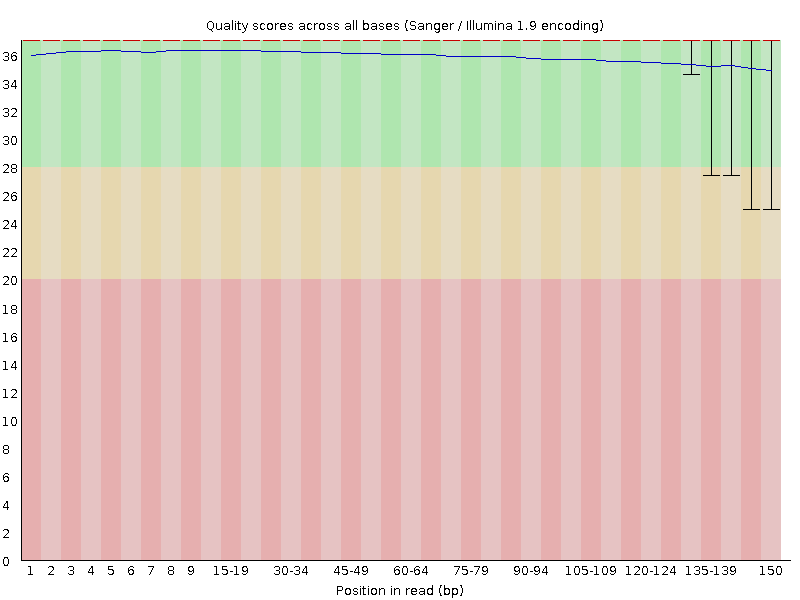

Supplement: Supplementary file 1 [file DataSheet1.ZIP › quality control/DF1_2/per_base_quality.png]

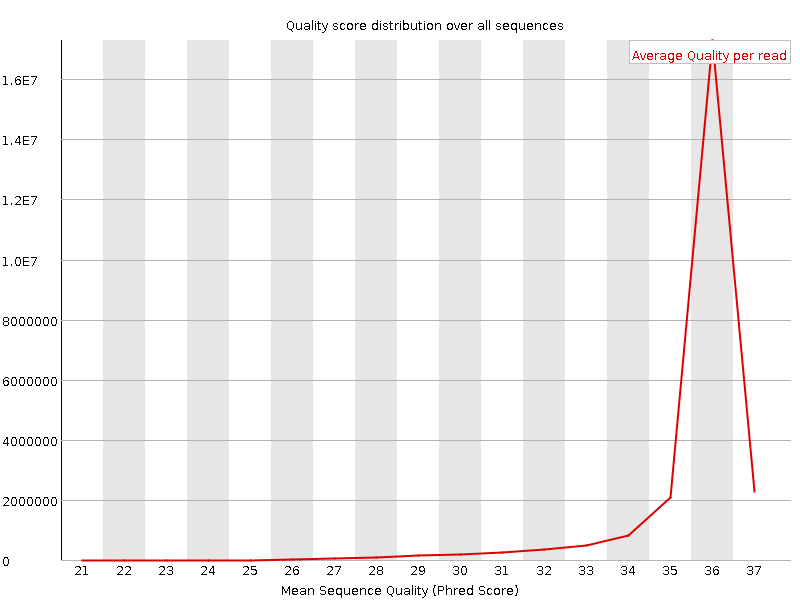

Supplement: Supplementary file 1 [file DataSheet1.ZIP › quality control/DF3_1/per_sequence_quality.png]

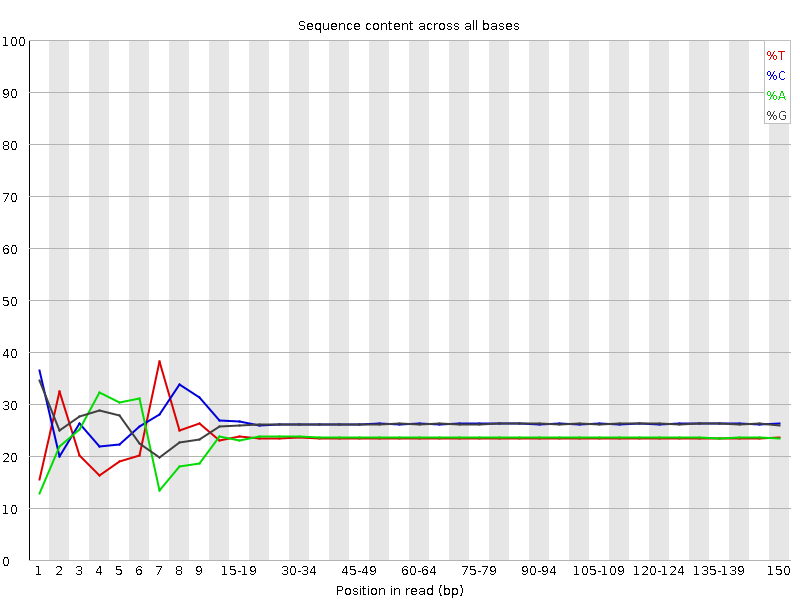

Supplement: Supplementary file 1 [file DataSheet1.ZIP › quality control/DF3_1/per_base_sequence_content.png]

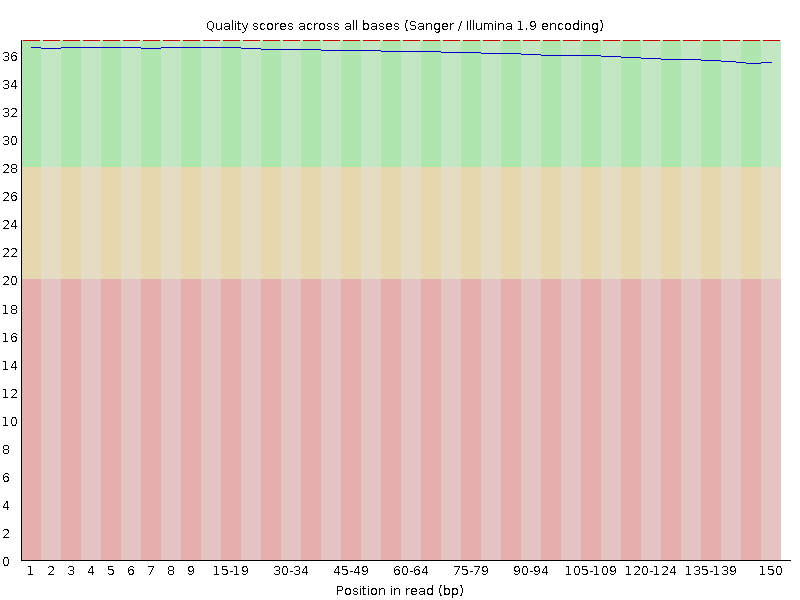

Supplement: Supplementary file 1 [file DataSheet1.ZIP › quality control/DF3_1/per_base_quality.png]
