# Supplementary material for: Integrating scRNA-seq and bulk RNA-seq to explore the differentiation mechanism of human nail stem cells mediated by onychofibroblasts
Source: Front Cell Dev Biol. 2024 Jun 3;12:1416780. doi: 10.3389/fcell.2024.1416780 (PMC11181305; doi:10.3389/fcell.2024.1416780)
Supplement: Supplementary file 2 [file DataSheet2.docx]

Supplementary Figures and Tables


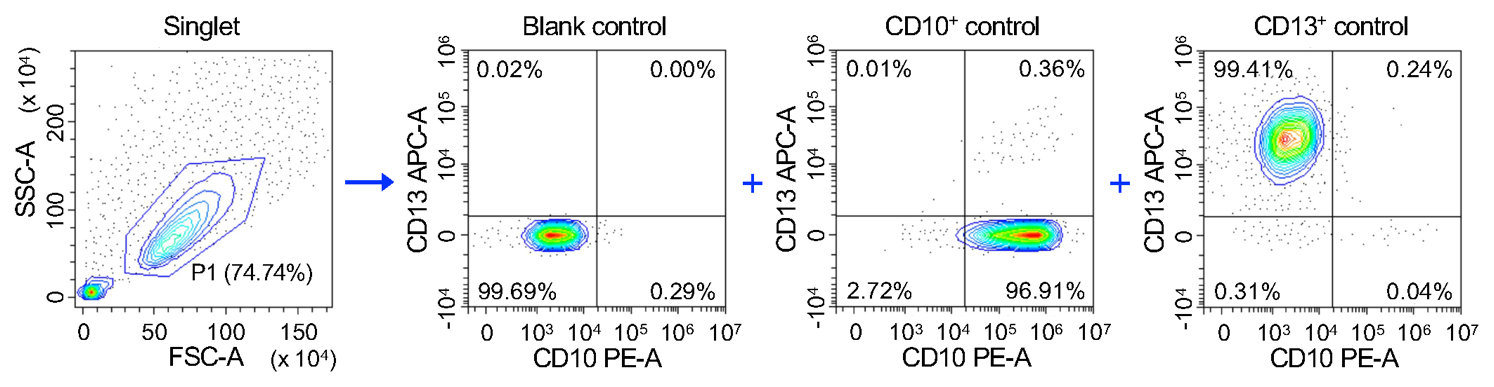


**Supplementary Figure 1.** **The gating strategy of CD10^+^ CD13^+^ human onychofibroblasts.**


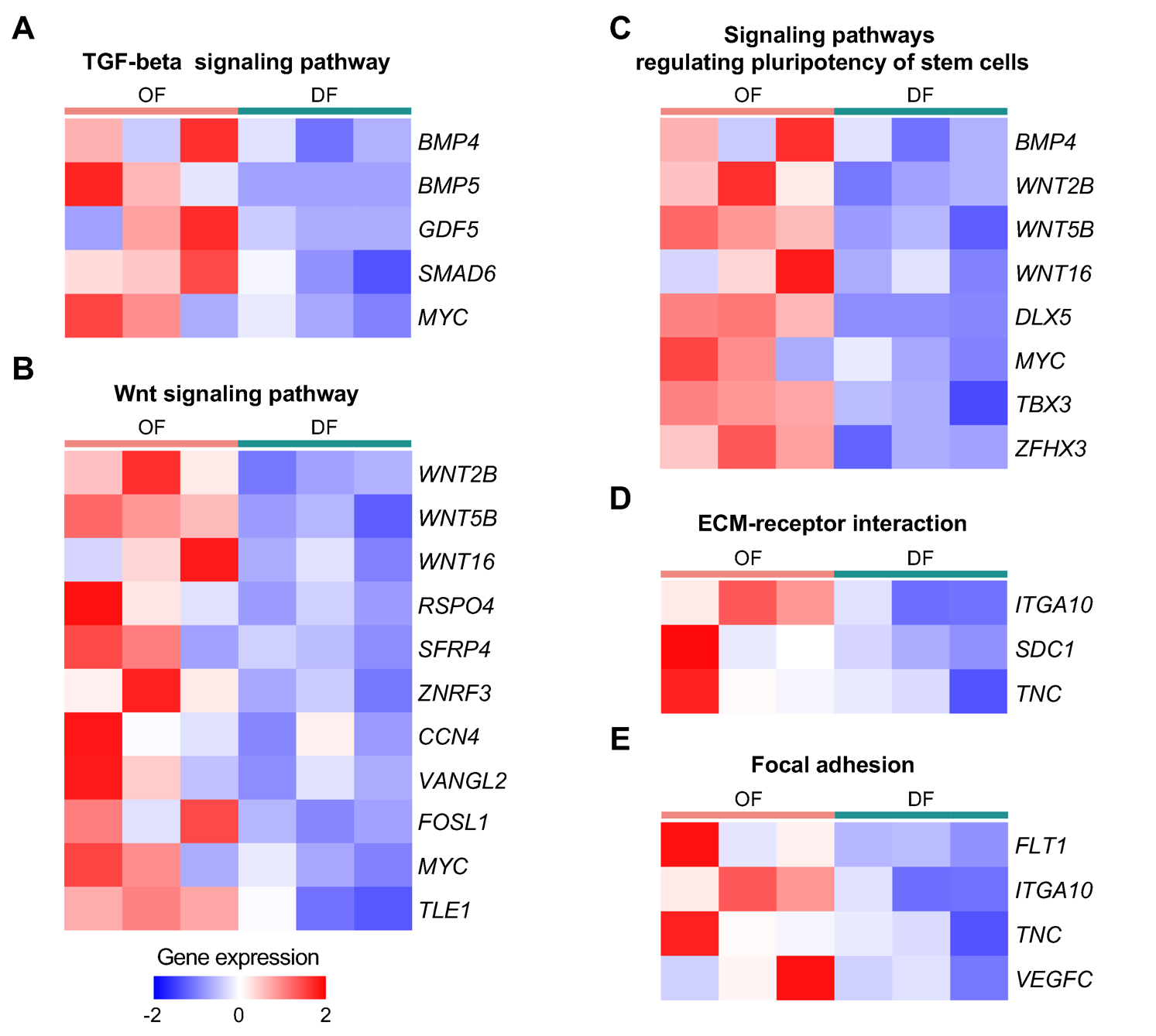


**Supplementary Figure 2.** **KEGG pathway analysis identified upregulated transcripts enriched in human onychofibroblasts.** Heatmaps of selected genes from **(A)** TGF-beta, **(B)** Wnt, **(C)** regulation of stem cell pluripotency, **(D)** ECM-receptor interaction, and **(E)** focal adhesion pathways upregulated in OFs compared to DFs.


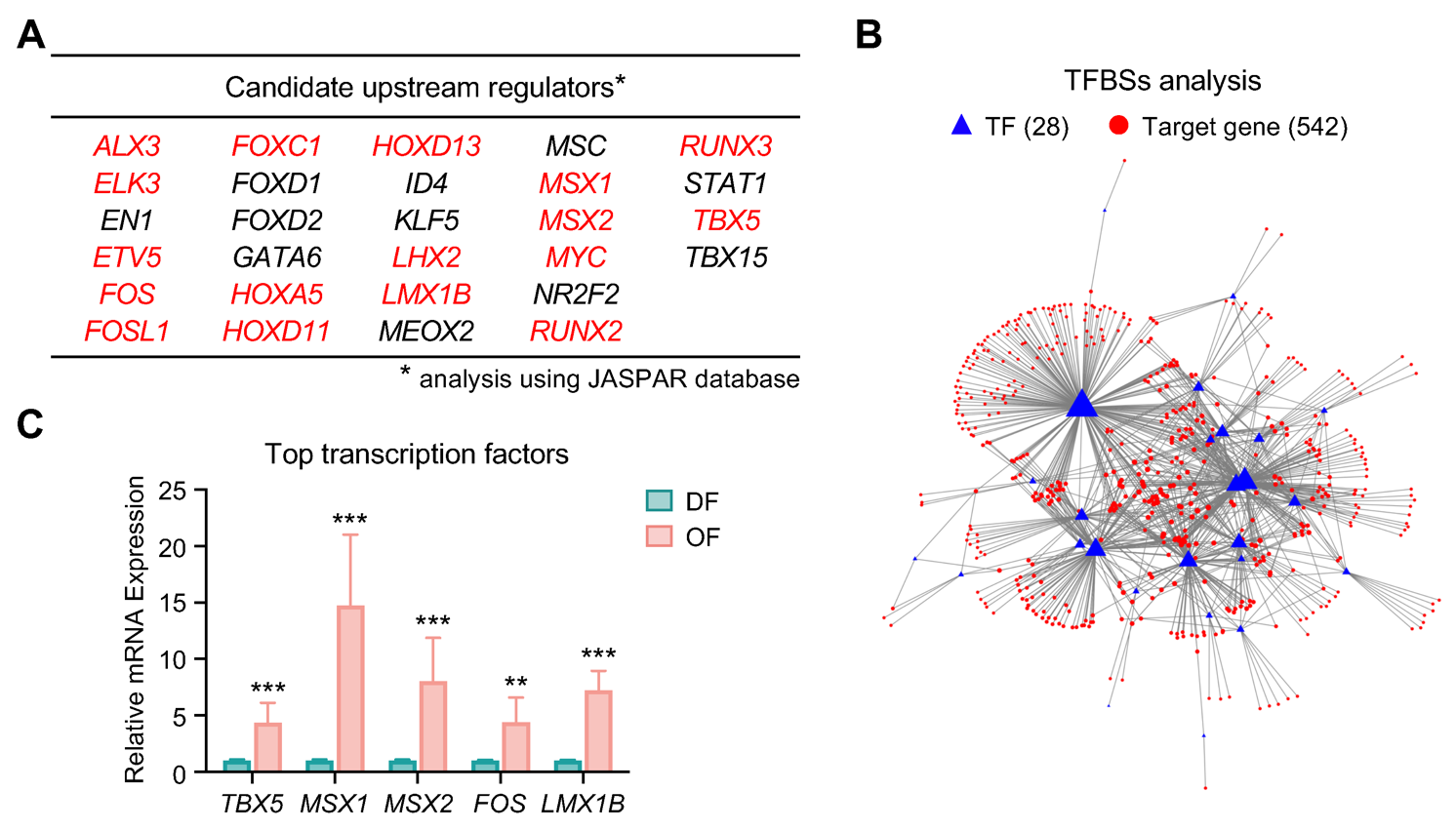


**Supplementary Figure 3.** **Identification of key transcription factors regulating differentially expressed genes.** **(A)** TFs predicted by the JASPAR database. The genes highlighted in red denote TFs upregulated in OFs. **(B)** Network illustrating the interaction between TFs and their target genes. Blue nodes represent TF genes, while red nodes represent target genes. **(C)** Relative mRNA expression levels of the top five promising hub TFs in DF and OF groups. The results presented are the mean ± SD of three biologically independent replicates. ** *P*＜0.01, *** *P*＜0.001.


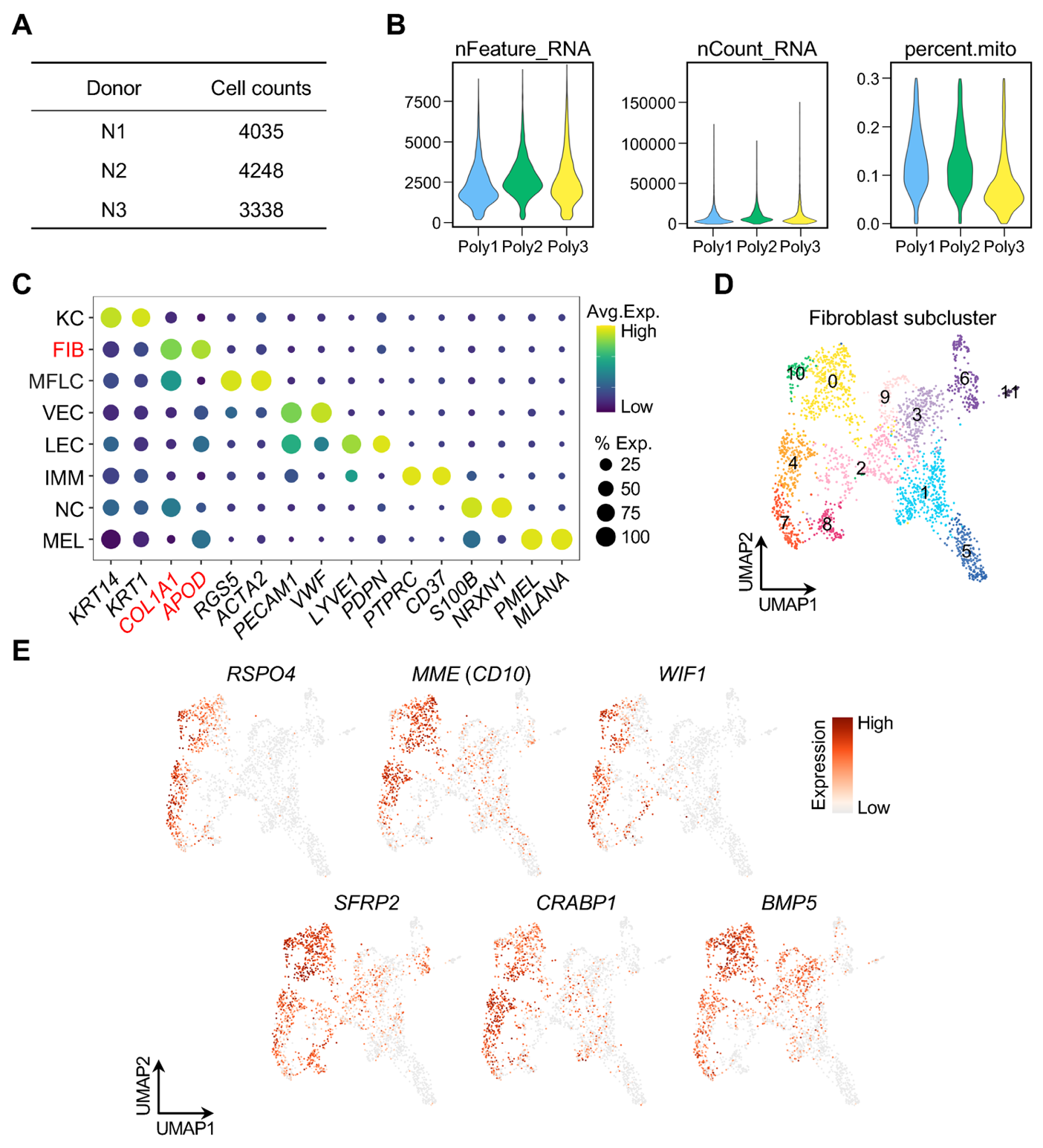


**Supplementary Figure 4.** **Summary of scRNA-seq dataset used in the analysis.** **(A)** Sample information and cell counts of each dataset. **(B)** quality control metrics for scRNA-seq data (200 < nFeature_RNA < 10000, percent.mito ≤ 30). nFeature_RNA, number of gene features; nCount_RNA, number of gene counts; percent.mito, percent of mitochondria. **(C)** Dot plot illustrating expression of cell state markers. The color key from purple to yellow represents low to high gene expression levels, and the dot size indicates the percentage of cells expressing the gene. KC, keratinocyte; FIB, fibroblast; MFLC, myofibroblast-like cell; VEC, vascular endothelial cell; LEC, lymphatic endothelial cell; IMM, immune cell; NC, neural cell; MEL, melanocyte; Exp, expression. **(D)** UMAP visualization of the fibroblast subcluster. **(E)** UMAP plot displaying expression of representative marker genes of OFs. The color intensity indicates gene expression levels.


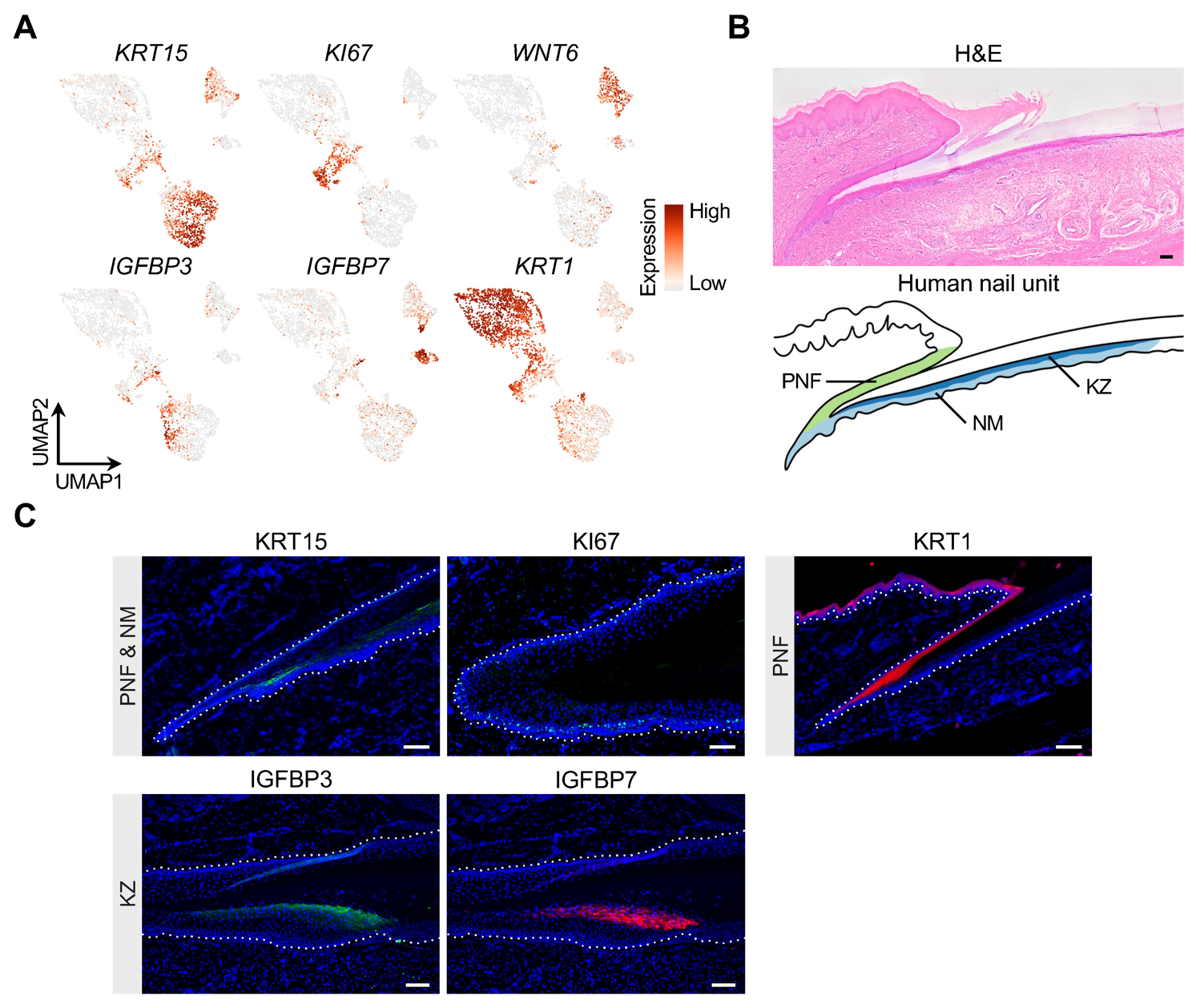


**Supplementary Figure 5.** **Main keratinocyte subtypes in human nail epithelium.** **(A)** UMAP plot illustrating the expression of marker genes for each keratinocyte subtype. The color key from gray to red indicates low to high gene expression levels. **(B)** Tissue compartments of the human nail epithelium. **(C)** Immunofluorescence staining depicts the localization of each keratinocyte subtype in the human nail epithelium. The dotted line delineates the boundary between the epidermis and dermis. PNF, proximal nail fold; NM, nail matrix; KZ, keratogenous zone. Scale bars, 100 μm.


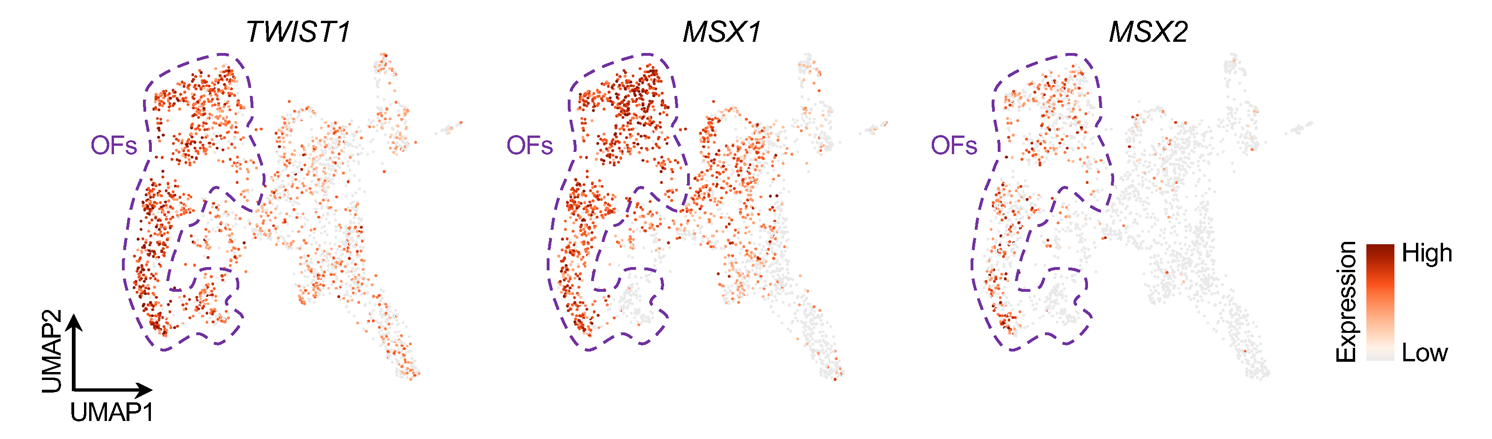


**Supplementary Figure 6.** **Key transcription factors of dermal stem cells are highly expressed in the onychofibroblast population revealed by scRNA-seq.** UMAP plot illustrates the expression of relevant transcription factor genes in OFs. The dotted area represents the OF population. OF, onychofibroblast. The color intensity indicates gene expression levels.

**Supplementary Table 1. Clinical Characteristics of Patients**

| **Order** | **Sex** | **Age (m)** | **Affected hand** | **bulk RNA-seq** | **Cell isolation** | **Histological staining** |
| --- | --- | --- | --- | --- | --- | --- |
| N1 | Female | 9 | Right | Yes | Yes | — |
| N2 | Male | 10 | Right | Yes | Yes | — |
| N3 | Male | 12 | Right | Yes | Yes | — |
| N4 | Male | 12 | Left | — | Yes | — |
| N5 | Female | 12 | Right | — | Yes | — |
| N6 | Male | 14 | Left | — | Yes | — |
| N7 | Female | 16 | Left | — | Yes | — |
| N8 | Male | 20 | Right | — | Yes | — |
| N9 | Female | 21 | Right | — | Yes | — |
| N10 | Male | 12 | Left | — | Yes | — |
| N11 | Female | 8 | Left | — | — | Yes |
| N12 | Female | 11 | Right | — | — | Yes |
| N13 | Male | 12 | Right | — | — | Yes |
| N14 | Male | 14 | Right | — | — | Yes |
| N15 | Male | 15 | Left | — | — | Yes |
| N16 | Female | 18 | Left | — | — | Yes |
| N17 | Female | 16 | Right | — | — | Yes |

**Supplementary Table 2. Antibodies List**

| **Antibodies** | **Host** | **Usage** | **Dilution** | **Supplier** | **Cat.no** |
| --- | --- | --- | --- | --- | --- |
| ***Primary antibodies*** |  | | | | |
| BMP4 | Rabbit | WB | 1:2000 | Abcam | ab39973 |
|  | | ICC | 1:300 |  | |
| Phospho-SMAD1/5/9 | Rabbit | WB | 1:2000 | Cell Signaling Technology | 13820 |
|  | | ICC | 1:300 |  |  |
| SMAD4 | Rabbit | ICC | 1:300 | Cell Signaling Technology | 46535 |
| GAPDH | Mouse | WB | 1:3000 | Proteintech | 60004-1-Ig |
| KRT15 | Mouse | ICC | 1:200 | Abcam | ab80522 |
|  | | IF | 1:200 |  | |
|  | | Flow | 1:100 |  | |
| KRT1 | Rabbit | IF | 1:400 | Abcam | ab185628 |
|  | Mouse | Flow | 1:100 | Invitrogen | MA1-06312 |
| KI67 | Mouse | IF | 1:200 | Cell Signaling Technology | 9449 |
| IGFBP3 | Mouse | IF | 1:100 | R&D Systems | MAB305 |
| IGFBP7 | Rabbit | IF | 1:200 | Abcam | ab171085 |
|  | | ICC | 1:200 |  | |
| CD10 (PE) | Mouse | Flow | 1:100 | BD Pharmingen | 561002 |
| CD13 (APC) | Mouse | Flow | 1:100 | BD Pharmingen | 561698 |
| CD29 (FITC) | Mouse | Flow | 1:200 | Invitrogen | 11-0299-42 |
| CD31 (FITC) | Mouse | Flow | 1:100 | BD Pharmingen | 555445 |
| CD34 (PE) | Mouse | Flow | 1:200 | Invitrogen | 12-0349-42 |
| CD44 (PE) | Rat | Flow | 1:200 | Invitrogen | 12-0441-82 |
| CD90 (FITC) | Mouse | Flow | 1:200 | Invitrogen | 11-0909-42 |
| CD105 (PE) | Mouse | Flow | 1:200 | Invitrogen | 12-1057-42 |
| ***Secondary antibodies*** |  | | | | |
| Peroxidase-AffiniPure™ Goat Anti-Rabbit IgG (H+L) | Goat | WB | 1:8000 | Jackson | 111-035-003 |
| Peroxidase AffiniPure™ Goat Anti-Mouse IgG (H+L) | Goat | WB | 1:8000 | Jackson | 115-035-003 |
| Donkey Anti-Mouse IgG H&L (Alexa Fluor® 488) | Donkey | IF | 1:1000 | Abcam | ab150105 |
|  |  | Flow | 1:2000 |  |  |
| Donkey Anti-Rabbit IgG H&L (Alexa Fluor® 594) | Donkey | 1F | 1:1000 | Abcam | ab150076 |

**Supplementary Table 3.** **Primers List**

| **Gene** | **Forward primer (5’-3’)** | **Reverse primer (5’-3’)** |
| --- | --- | --- |
| *PPAR-γ* | TTCCAACTCCCTCATGGCAA | GTTCTCCGGAAGAAACCCTTG |
| *GLUT4* | CGACCAGCATCTTCGAGACA | CACCAACAACACCGAGACCA |
| *FABP4* | GGGCCAGGAATTTGACGAAG | ACGCATTCCACCACCAGTT |
| *LEP* | GACATTTCACACACGCAGTC | ATAAGGTCAGGATGGGGTGGA |
| *LPL* | ACACTTGCCACCTCATTCCC | ACATTCCTGTTACCGTCCAGC |
| *ALP* | ACCGAGATACAAGCACTCCC | CTCGTACTGCATGTCCCCTG |
| *RUNX2* | TGTCATGGCGGGTAACGATG | GTGAAACTCTTGCCTCGTCC |
| *IBSP* | AACGATTTCCAGTTCAGGGCA | AGCCCAGTGTTGTAGCAGAA |
| *OCN* | GCCACCGAGACACCATGAGAG | GGGTCTCTTCACTACCTCGCT |
| *OPN* | AGACCTGACATCCAGTACCCT | CAACGGGGATGGCCTTGTAT |
| *SOX9* | GGAAGTCGGTGAAGAACGGG | CCTTGAAGATGGCGTTGGGG |
| *COL II* | GAAGGATGGCTGCACGAAAC | TCCACACCGAATTCCTGCTC |
| *ACAN* | AGGAGATGGAGGGTGAGGTC | CACTCATTGGCTGCTTCCTG |
| *TBX5* | CAGAACCACAAGATCACGCA | TGGGGACCACGGGATATTCT |
| *MSX1* | AAAGTGGCTGGAAGAGTCCC | ACACCGATTTCTCTGCGCTT |
| *MSX2* | AAGGCGAAAAGACTGCAGGA | GCTGATGGGGAAAGGGAGAC |
| *FOS* | GGGGCAAGGTGGAACAGTTAT | AGGTTGGCAATCTCGGTCTG |
| *LMX1B* | TCCTGATGCGAGTCAACGAG | CCGCGAAGAGCTGTTGGTA |
| *BMP4* | AGCTTCCACCACGAAGAACAT | AAGCCCCTTTCCCAATCAGG |
| *FGF10* | GGGGAAACTCTATGGCTCAAAAG | CCTCCCATTATGCTGCCAGT |
| *KRT15* | CCATCAGGGAAGCCTCTTCA | CCACCTGTCCATCCACTGAC |
| *KI67* | TGGGCACCTAAGACCTGAACT | ATGGTTGAGGCTGTTCCTTGATGA |
| *WNT6* | CGGGGAGCGTTTAAAGGACA | TTATTGATACTAACCTCACCCACC |
| *IGFBP3* | GTTCCACCCCCTCCATTCAA | CTCTACGGCAGGGACCATATTC |
| *IGFBP7* | AGCTGTGAGGTCATCGGAATC | ACTTAGAGGAGATACCAGCACCC |
| *KRT1* | ATGGACAACAACCGCAGTCT | TGCAGCTCTTCATACTTGCTCT |
| *18S* | CCAGACAAATCGCTCCACCAAC | GACTCAACACGGGAAACCTCAC |
